# Machine Learning-Driven Prediction and Interactive Nonlinear Analysis of Compaction Parameters for Fine-Grained Soils

**DOI:** 10.3390/ma19173717

**Published:** 2026-08-31

**Authors:** Hongwei Wang, Hui Ye, Tingting Zhao, Daoyuan Sun, Fang Yan

**Affiliations:** 1 School of Resources and Safety Engineering, Central South University, Changsha 410083, China; hongwei.wang@csu.edu.cn (H.W.);; 2 State Key Laboratory of Metal Mining Safety and Disaster Prevention and Control, Changsha 410083, China

**Keywords:** fine-grained soil, compaction parameters, WHO, machine learning, SHAP interpretability, PDPs

## Abstract

Compaction parameters of soil material, maximum dry density (MDD) and optimum moisture content (OMC), are critical control indicators for highway embankment construction. In this study, a dataset containing 199 compaction test results for fine-grained soils was collected. Using MDD and OMC as prediction targets, Random Forest (RF), Gradient Boosting (GB), Extreme Gradient Boosting (XGBoost), and Support Vector Regression (SVR) were developed and optimized using the Wild Horse Optimization (WHO) algorithm. Five universal evaluation metrics, combined with radar charts, were used to comprehensively compare the predictive performance of each model. Based on univariate sensitivity analysis and SHapley Additive exPlanations (SHAP) global feature interpretation, two-way partial dependence plots (PDPs) were applied to reveal the pairwise nonlinear relationships between soil physical indices and compaction indicators. The results demonstrate that WHO-tuned XGBoost achieves optimal comprehensive predictive performance for both MDD (test set R^2^ = 0.8226) and OMC (test set R^2^ = 0.7486), outperforming SVR, GB, and RF in terms of fitting accuracy and generalization under small-sample conditions. Plastic limit (PL) exerts a significant influence on compaction performance. By further comparing WHO, Particle Swarm Optimization (PSO), Gray Wolf Optimizer (GWO), Random Search, Bayesian Optimization and Grid Search on the optimal model, the applicability and feasibility of WHO-XGBoost in predicting compacted-soil compaction parameters were validated. The proposed data-driven compaction evaluation framework (WHO-XGBoost-SA-SHAP-PDPs) acts as an auxiliary tool to lower the workload and cost of laboratory Proctor tests, offering theoretical support and technical guidance for rapid refined embankment compaction control in green transportation infrastructure.

## 1. Introduction

Natural soil is the most widely used granular geotechnical material for the backfill of embankments and retaining structures in sustainable transportation engineering [1,2,3,4]. Compaction technology is a core technical measure to improve soil mechanical properties and ensure road performance [5,6]. Under external rolling loads, soil particles are extruded and rearranged, the internal pore volume is continuously compressed, and the interlocking engagement and contact friction between particles are greatly enhanced, eventually forming a denser and more stable soil structure [7,8,9]. Maximum dry density (MDD) and optimum moisture content (OMC) are core characteristic parameters for evaluating soil compaction quality [10]. They can objectively reflect the compaction-forming condition of compacted soil and serve as critical control indicators to determine whether compacted soil meets the specification requirements. These two parameters directly determine the bearing capacity and water stability of embankments and exert a decisive influence on long-term service performance, such as settlement and differential deformation of roads [11]. At present, the measurement of compaction parameters of compacted soil mainly relies on laboratory Proctor compaction tests, including standard Proctor compaction and modified Proctor compaction. This test consumes a large number of undisturbed soil samples, and data are measured through layered sample preparation and multiple parallel tests [12,13,14]. In general, at least six samples need to be appropriately prepared to identify the compaction curve with reasonable accuracy. The full testing cycle for a single batch of samples can take 2 to 3 days, accompanied by high labor, material, and time costs. For large-scale expressway backfilling projects involving filler screening, classification, and optimal mix proportion selection, conventional laboratory tests are inefficient and fail to meet the engineering demands of rapid on-site construction and fast filler evaluation [15]. Furthermore, traditional linear fitting formulas and conventional multiple regression analysis cannot accurately characterize the complex nonlinear relationships between compaction performance and multiple factors, including particle fraction proportions, Atterberg limits, plasticity indices, and compaction energy, leading to poor adaptability and stability when predicting compaction parameters across various soil types [16,17]. Wang et al. [17] developed multiple linear regression (MLR) models to predict maximum dry density and optimum moisture content of solidified dredged sediments. Although subdividing the dataset into sandy and silty subsets slightly improved model accuracy, the derived linear equations failed to characterize the interactive effects of particle gradation, organic matter content and binder dosage on compaction parameters. Khuntia et al. [18] used multivariate adaptive regression splines (MARSs) to predict soil compaction parameters, yet the model captured only simple linear correlations between input variables and failed to describe complex nonlinear relationships between particle composition and plasticity indicators with values lower than 0.70. Kurnaz and Kaya [19] established an extreme learning machine (ELM) prediction framework based on 451 groups of standard Proctor test data; although ELM outperformed SVM and GMDH in fitting speed, the study reported only single-variable correlation coefficients and did not reveal the interactive effects of multiple soil indices on compaction performance. Wang and Yin [20] built an MEP model using 226 groups of soil data from published papers, but the research merely reported global prediction errors and ignored how plastic limit and sand fraction jointly alter MDD and OMC. Jalal et al. [21] separately developed GEP and MEP models for expansive soil compaction prediction and evaluated model superiority solely using error indicators, without conducting a two-factor joint-response analysis to disentangle synergistic or antagonistic feature interactions. Systematic investigations on the synergistic laws of multiple parameters are also scarce. Conventional single-factor sensitivity analysis and feature analysis can only reveal the independent marginal effect of a single variable but fail to describe the dynamic evolution laws of compaction indicators under the joint action of two variables. As a result, existing research findings have obvious limitations in guiding on-site embankment compaction construction.

To address the above deficiencies, this study integrates 199 sets of test data of fine-grained soil (particle size < 2 mm). Taking maximum dry density (MDD) and optimum moisture content (OMC) as dual prediction targets, four machine learning models, including Random Forest (RF), Gradient Boosting (GB), Extreme Gradient Boosting (XGBoost), and Support Vector Regression (SVR), are optimized via the Wild Horse Optimization (WHO) algorithm. The optimal model is determined through comprehensive performance comparison. Based on feature importance and sensitivity analysis, two-way partial dependence analysis is employed to reveal the nonlinear variation in compaction indicators under parameter coupling, overcoming the limitations of traditional single-factor methods. Further, the optimal model is fine-tuned using a variety of hyperparameter optimization approaches, and the applicability of different tuning strategies is systematically compared. The machine learning framework established in this study acts as an auxiliary prediction tool. For fills with soil characteristics similar to those in the dataset, the model can rapidly estimate compaction indicators to support preliminary engineering design and on-site pre-evaluation. It can reduce the number of laboratory Proctor compaction tests and cut experimental costs. Meanwhile, the framework provides a reliable theoretical basis and technical guidance for refined embankment compaction control and intelligent optimization of filling parameters.

## 2. Methods

**Figure 1:**
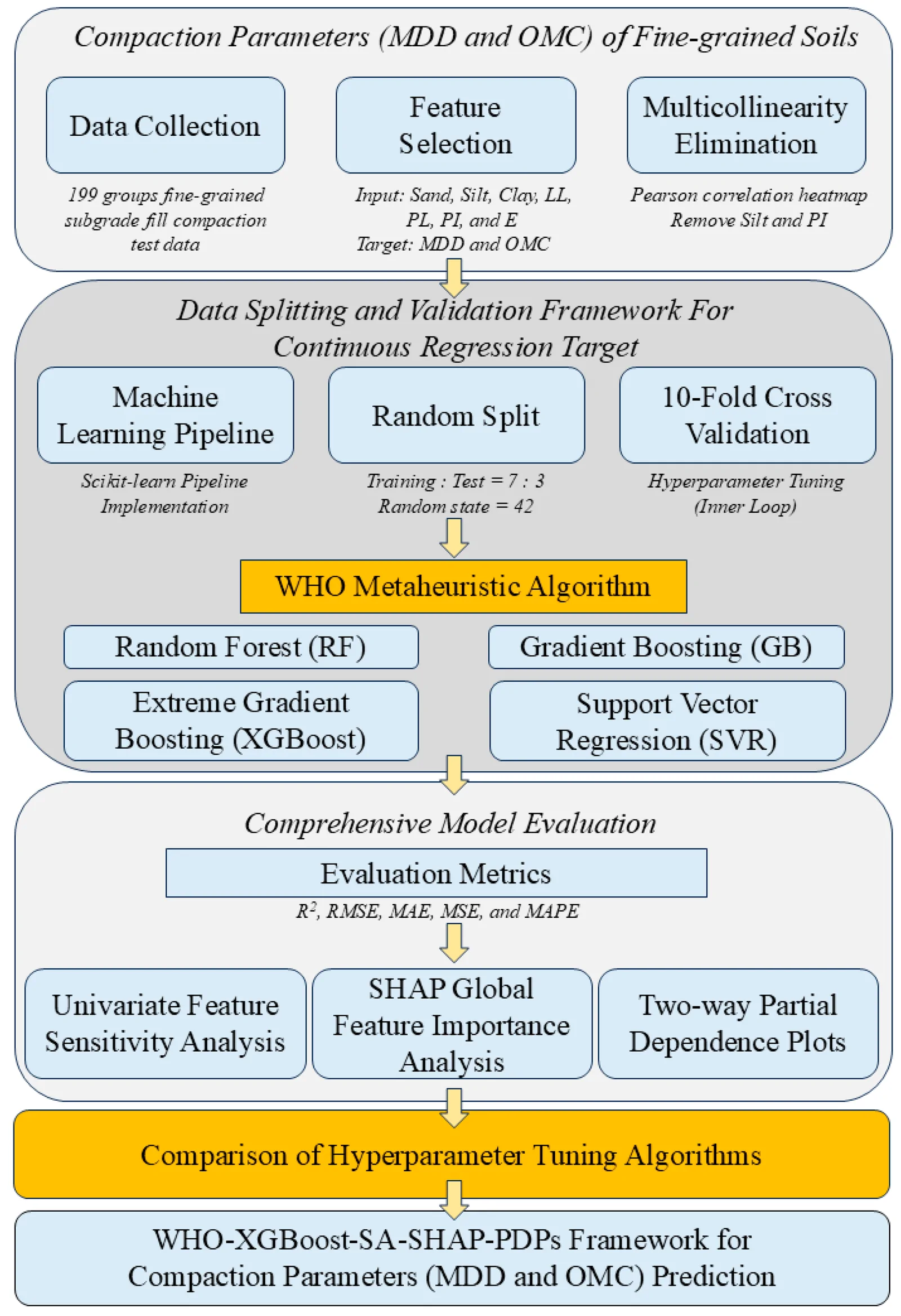
Development flowchart.

In this study, a machine learning workflow was established to predict the MDD and OMC of fine-grained soil. The detailed development procedure is shown in Figure 1.

Firstly, 199 groups of laboratory compaction test data of fine-grained soil were collected. Soil physical indices including sand content (sand), silt content (silt), clay content (clay), liquid limit (LL), plastic limit (PL), plastic index (PI), compaction energy (E), MDD and OMC were included in the database. The Pearson correlation heatmap was adopted to identify and eliminate multicollinear features to avoid model bias caused by redundant variables.

Subsequently, a unified machine learning pipeline based on Scikit-learn was constructed. The dataset was randomly divided into training and test sets at a 7:3 ratio, with a fixed random state of 42. A 10-fold cross-validation framework was adopted as the inner loop for hyperparameter tuning. Four representative ensemble and regression models, namely RF, GB, XGBoost and SVR, were established. The WHO metaheuristic algorithm was utilized to search for the optimal hyperparameter combination for each model.

After model training, comprehensive quantitative evaluation was implemented using five statistical indicators. On the basis of optimal models, interpretable mechanism analysis was further carried out. Univariate feature sensitivity analysis was performed to reveal the independent influence of each input variable. SHAP global feature importance analysis quantified the contribution ranking of each feature. Two-way partial dependence plots (PDPs) were adopted to characterize the marginal effects and interaction behaviors between different features. Finally, the performance of different hyperparameter tuning algorithms was compared to explore the optimization efficiency for regression models of embankment compaction parameters. Standardization was applied to all data throughout the entire workflow.

The entire development process was carried out in a Python 3.14 (64-bit) environment. The hardware configuration of the MagicBook 16 Pro (2021) laptop (Honor Device Co., Ltd., Shenzhen, China) includes an AMD Ryzen 7 5800H processor paired with Radeon Graphics (3.20 GHz), 16.0 GB memory (15.4 GB available), a 64-bit operating system, and an x64-architecture processor.

### 2.1. Data Collection and Processing

Through literature retrieval and screening, a total of 199 sets of experimental data on soil compaction parameters were collected in this study [22,23,24,25,26,27,28,29,30,31,32,33,34,35,36,37,38,39,40,41,42,43,44,45,46,47,48,49,50,51,52,53,54,55,56]. After unit standardization, these data from various literature sources were integrated into a new database to investigate the applicability of machine learning methods.

#### 2.1.1. Descriptive Statistics of the Database

Descriptive statistical analysis of the database enables intuitive observation of data distribution, and the resulting insights can be compared with machine learning predictions to validate the soundness of the modeling procedure. A total of 199 groups of cleaned experimental data were analyzed descriptively and statistically to clarify the distribution characteristics of soil particle fractions, plasticity indices, and compaction parameters. Table 1 presents the descriptive statistics results for the database. The particle composition analysis revealed that the tested samples were mainly silty soils, with silt accounting for the largest proportion, averaging 45.93%. Sand content varied drastically from 0.00% to 89.00%. Clay content had a moderate distribution, with an average of 28.50% and a range of 2.00% to 69.00%. LL and PI exhibited prominent dispersion, with maximum values of 510.00% and 480.00, respectively, indicating that the dataset encompassed soils with low to ultra-high plasticity. PL was distributed within a narrow span from 6.10% to 50.00% with a small standard deviation. Compaction energy E ranged from 155 kJ/m^3^ to 2700 kJ/m^3^ and possessed the maximum dispersion, representing various standard and heavy compaction efforts in geotechnical tests. By comparison, OMC (19.28% on average) and MDD (1.68 Mg/m^3^ on average) had narrow fluctuation ranges and low standard deviations. The stable distribution of these two critical compaction indices verified the rationality and representativeness of the established database, laying a solid foundation for the subsequent construction and validation of machine learning prediction models for soil compaction performance.

**Figure 2:**
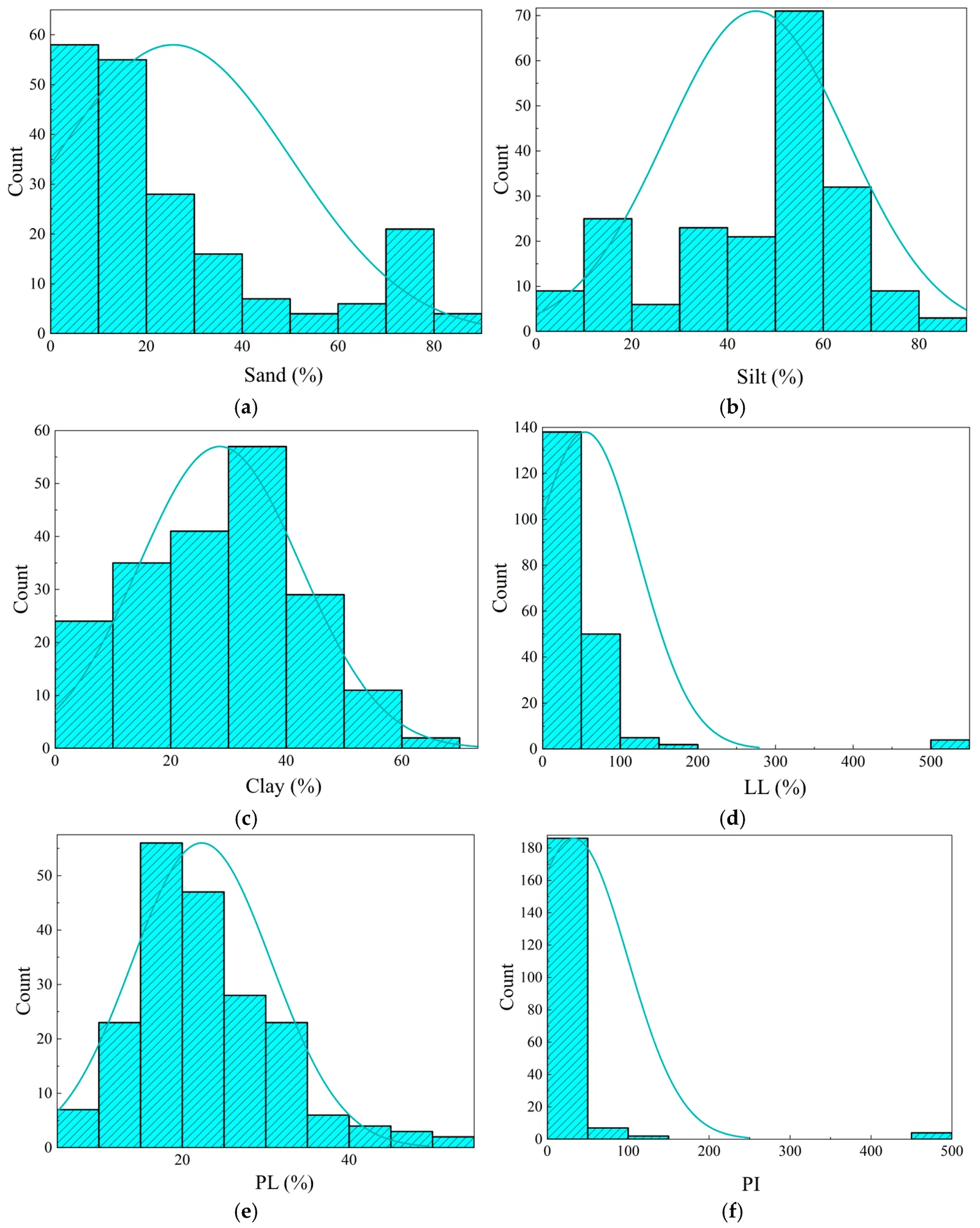

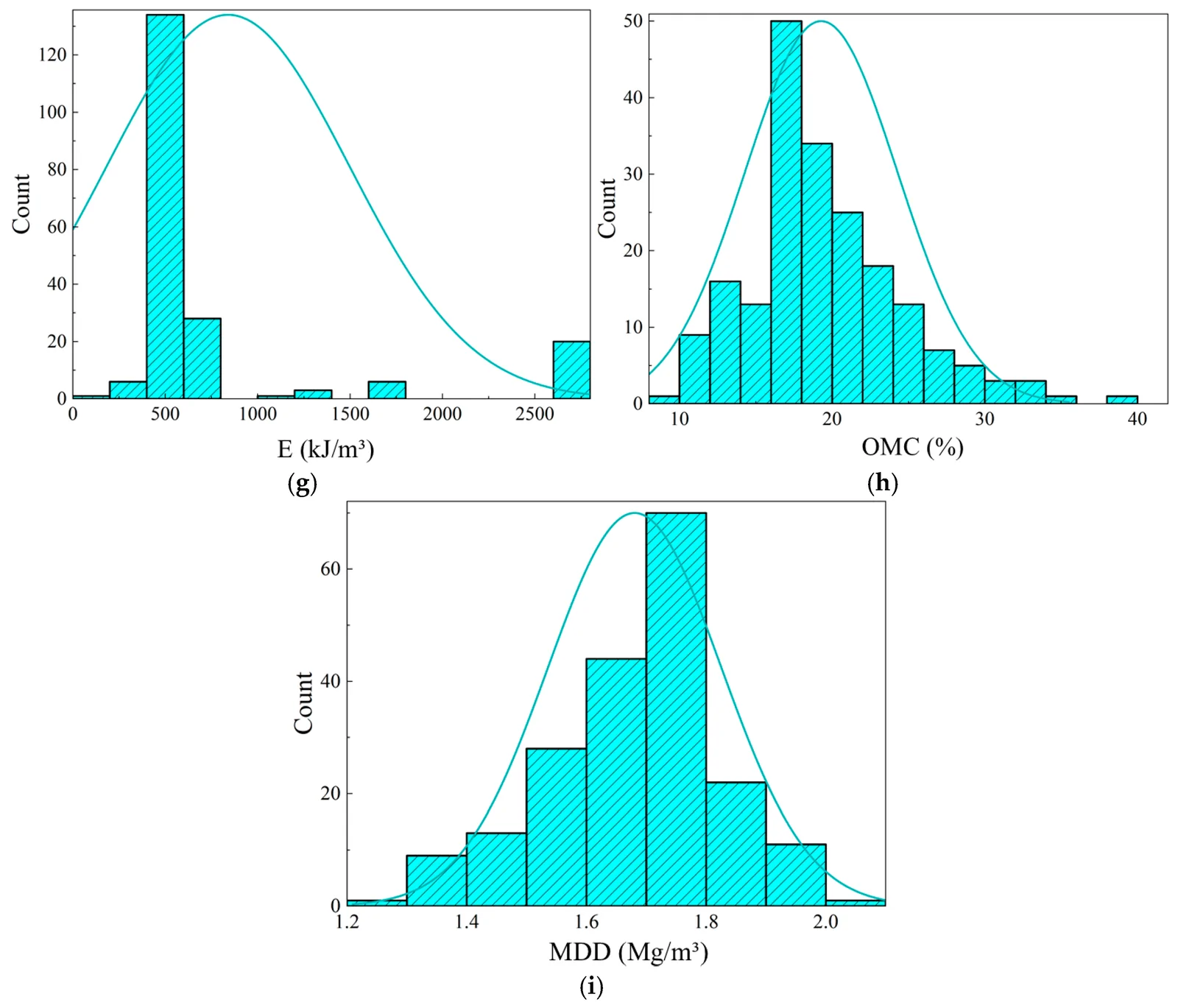
Frequency distributions of the soil properties in the dataset: (**a**) sand; (**b**) silt; (**c**) clay; (**d**) LL; (**e**) PL; (**f**) PI; (**g**) E; (**h**) OMC; and (**i**) MDD.

Figure 2 presents frequency histograms overlaid with fitted normal distribution curves for all variables in the soil compaction dataset. Silt, clay, plastic limit, OMC and MDD follow approximately symmetric normal distributions. Sand content is strongly right-skewed, with a peak at 0–20% and a long tail extending up to 89%. LL and PI also exhibit obvious positive skewness, with most data below 100 and 50, and only sporadic extremely high values. Compaction energy E shows the most prominent right skew, with peak frequency within 0–500 kJ/m^3^ and scarce data above 1000 kJ/m^3^. These diverse distribution patterns embody the natural variability of soil physical and mechanical properties. Particle fractions and plasticity indices mostly display skewed patterns controlled by inherent soil composition, while the two core compaction parameters, OMC and MDD, feature stable symmetric distributions, supporting the dataset’s reliability for subsequent machine learning modeling.

#### 2.1.2. Pearson Correlation Heatmap Analysis

**Figure 3:**
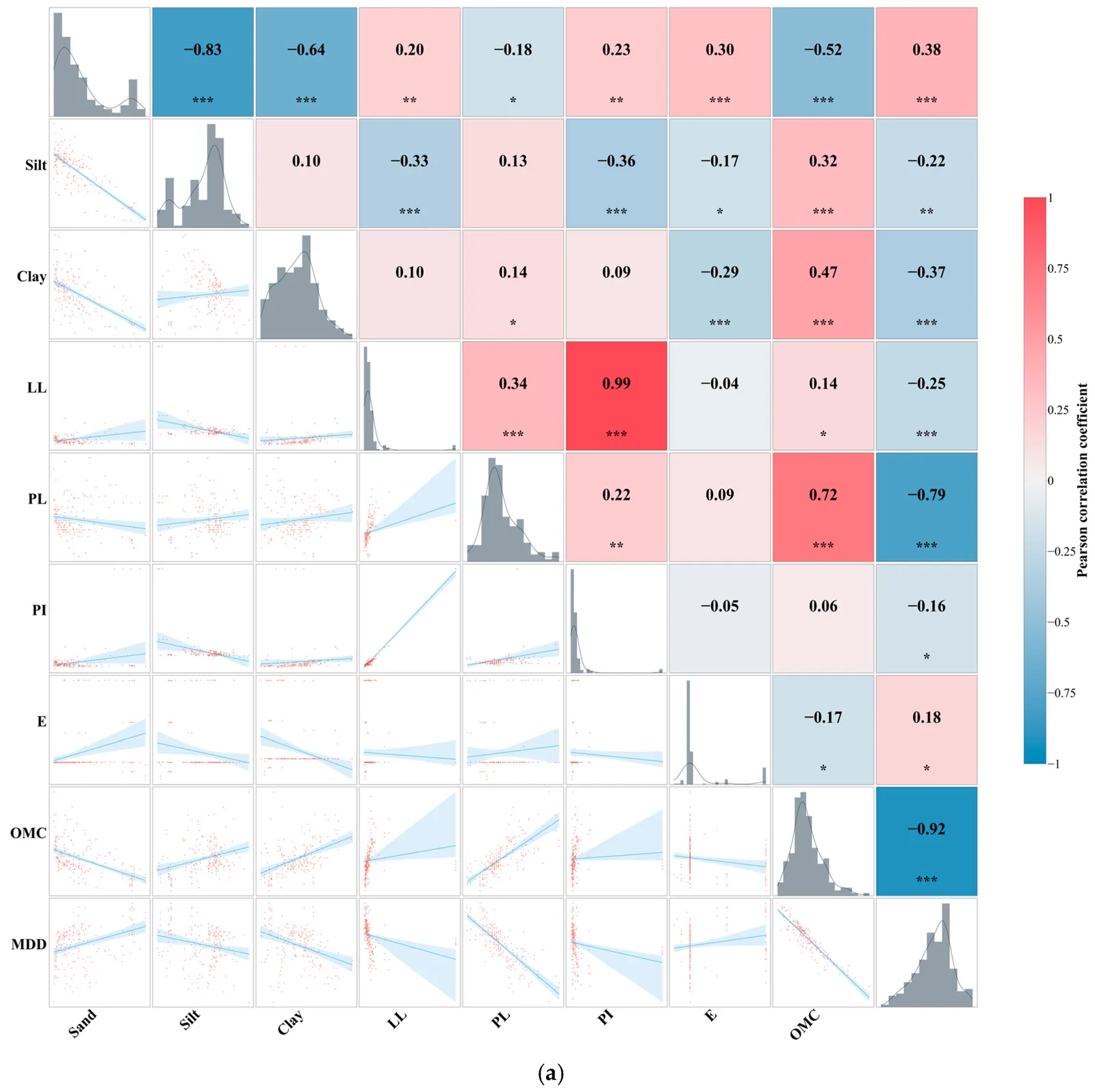

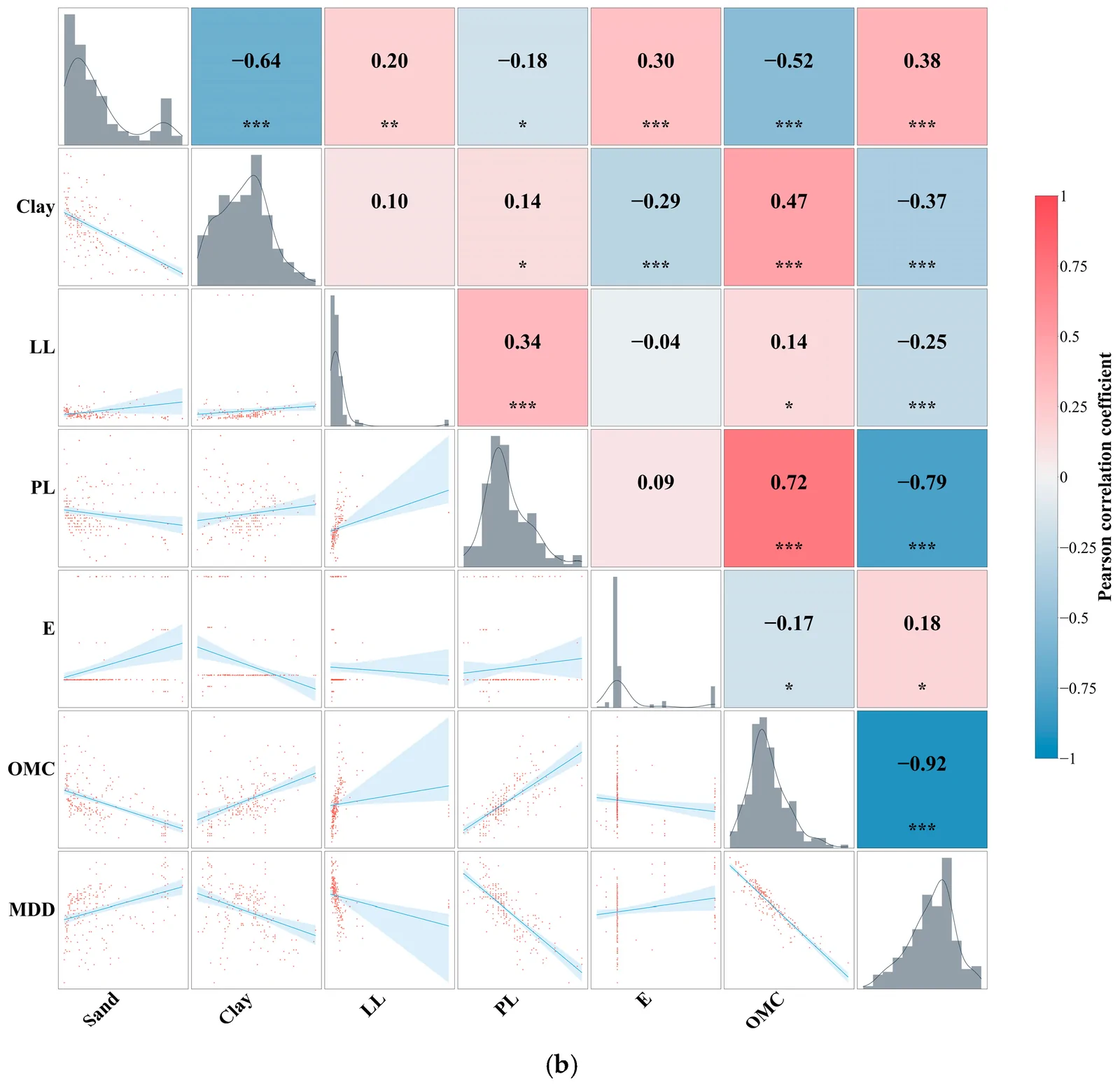
Results of Pearson correlation analysis: (**a**) before feature selection; (**b**) after feature selection. Significance levels are indicated as: * *p* < 0.05, ** *p* < 0.01, and *** *p* < 0.001.

In this study, Pearson correlation analysis was performed on the dataset to quantify the degree of correlation between variables and identify redundant features to reduce computational cost. This approach can effectively enhance the stability and generalization performance of the model and provide theoretical guidance for the subsequent feature selection process. Figure 3a presents the correlation results of the initial dataset. The results show that strong linear correlations exist between sand and silt as well as between PI and LL, with absolute correlation coefficients exceeding 0.8. Such high correlations may easily lead to multicollinearity, which is unfavorable for subsequent model construction. After careful consideration, silt and PI were removed from both variable groups, while sand and LL were retained as input features. Pearson correlation analysis was then performed again on the dataset after feature selection, as shown in Figure 3b. The preprocessed dataset shows no significant multicollinearity among input features.

### 2.2. Model Development

Based on a comprehensive literature review, the following four machine learning algorithms were selected for predictive modeling and comparative analysis in this study: Random Forest (RF), Gradient Boosting (GB), Extreme Gradient Boosting (XGBoost), and Support Vector Machine (SVM). RF is a bagging ensemble model with strong generalization and anti-overfitting ability. GB and XGBoost belong to boosting ensemble frameworks that iteratively minimize residuals to capture complex nonlinear relationships, and XGBoost further optimizes regularization and computational efficiency. SVM utilizes kernel functions to address high-dimensional and nonlinear fitting problems. Based on the same dataset, all models were trained and evaluated to systematically compare their predictive performance.

#### 2.2.1. Extreme Gradient Boosting (XGBoost)

XGBoost is a scalable tree ensemble system optimized and upgraded from Gradient Boosting Decision Tree (GBDT). This algorithm uses a second-order approximation of the loss function and applies dual L1 and L2 regularization constraints, effectively suppressing model overfitting at the algorithmic level. Meanwhile, it adopts a series of engineering strategies including dedicated computation logic for sparse data, weighted quantile sketch, cache optimization and block storage. These strategies enable parallel computation for node splitting and out-of-core computation, thereby greatly improving training speed and data processing capability.

#### 2.2.2. Support Vector Regression (SVR)

SVR is a classic supervised learning algorithm derived from statistical learning theory. It constructs an optimal hyperplane in the high-dimensional feature space to perform regression and numerical prediction, while maximizing the classification margin and minimizing the overall fitting error across samples. Combined with kernel functions, SVR can effectively capture the nonlinear mapping between input variables and target values and effectively solve nonlinear regression problems with high-dimensional data. It exhibits excellent generalization performance on small and medium-sized datasets and strong robustness against data noise and outliers, making it suitable for the nonlinear prediction analysis of geotechnical material parameters.

#### 2.2.3. Gradient Boosting (GB)

GB is a classic ensemble learning algorithm based on the boosting framework. It trains decision trees iteratively to continuously fit and correct the residuals of previous models and then integrates all base learners to generate predictions. This algorithm excels at capturing nonlinear relationships between variables and serves as the fundamental prototype of Extreme Gradient Boosting.

#### 2.2.4. Random Forest (RF)

RF is a classic ensemble machine learning algorithm based on the Bagging framework. It constructs multiple independent decision trees by randomly sampling training samples and features and then outputs predictions via voting or averaging. Compared with single models, Random Forest can effectively reduce the risk of overfitting and improve generalization performance. It also exhibits strong robustness to high-dimensional features and discretely distributed data and is widely applied in regression and predictive research.

#### 2.2.5. Wild Horse Optimizer (WHO)

**Figure 4:**
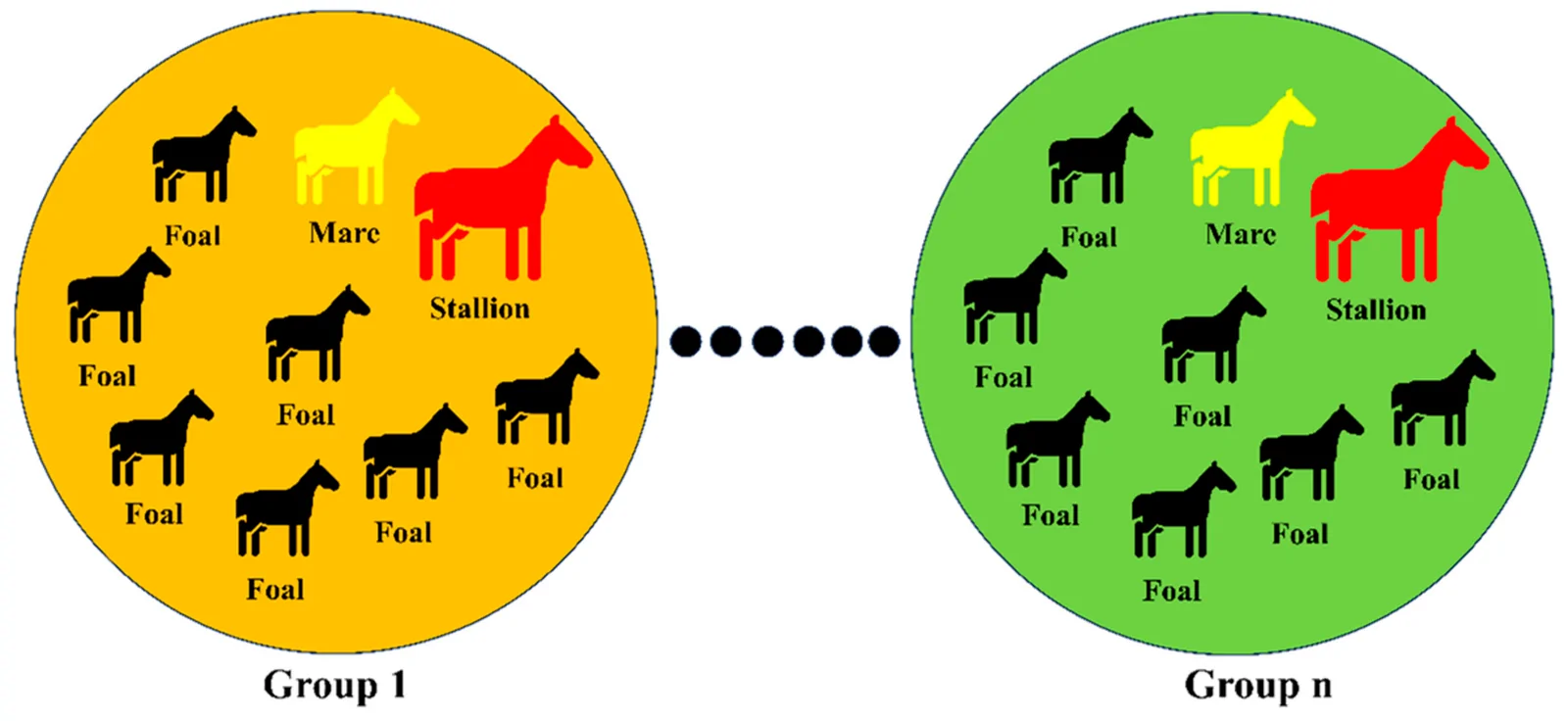
Formation of horse groups tailored to the initial population.

The Wild Horse Optimizer (WHO) is a metaheuristic algorithm inspired by the social behavior of wild horses. It divides the population into multiple groups led by stallions (Figure 4). Grazing behavior is used for global exploration, while inter-group mating realizes local exploitation. An adaptive parameter TDR is adopted to dynamically adjust the search range (Equation (1)).

(1) $$TDR=1-\frac{iter}{maxiter}$$

TDR is the adaptive step-size control parameter that linearly decreases from 1 to 0 as the iteration proceeds, iter denotes the current iteration, and maxiter denotes the maximum number of iterations.

Equation (2) simulates the grazing behavior of mares and foals around the stallion (group leader). ${\bar{X}}_{i,G}^{j}$ is the updated position of the ith member in the Gth group for the jth dimension. $X_{i,G}^{j}$ is its current position, and ${\mathrm{Stallion}}^{j}$ is the position of the group leader. R is a random number uniformly distributed in [−2, 2], and Z is an adaptive variable determined by TDR to adjust the search range.

(2) $${\overset{-}{X}}_{i,G}^{j}=2Z\cos(2\pi RZ)\times \left({\mathrm{Stallion}}^{j}-X_{i,G}^{j}\right)+{\mathrm{Stallion}}^{j}$$

Equation (3) represents the inter-group mating operation, which is designed to avoid inbreeding and maintain population diversity. $X_{G,K}^{p}$ is the new offspring generated by crossover. $X_{G,i}^{q}$ and $X_{G,j}^{z}$ are individuals selected from different groups, with indices i,j,k belonging to distinct groups. The crossover probability is typically set to PC = 0.13. The algorithm terminates when the maximum number of iterations is reached.

(3) $$X_{G,K}^{p}=\mathrm{Crossover}\left(X_{G,i}^{q},X_{G,j}^{z}\right),\;i\neq j\neq k$$

#### 2.2.6. Model Evaluation

To comprehensively evaluate the performance of the model, the indicators in Table 2 are selected for a comprehensive assessment. The closer the indicators are to the ideal values, the better the model performs. The coefficient of determination (R^2^) characterizes the consistency of variation trends between predicted and measured values. The root mean square error (RMSE) is more sensitive to significant prediction deviations and reflects the model performance for extreme samples. The mean absolute error (MAE) represents the overall average error and intuitively quantifies the average deviation of predicted results. The mean absolute percentage error (MAPE) eliminates dimensional differences, facilitating horizontal comparisons of prediction accuracy for compaction parameters with different magnitudes. Complementary joint evaluation using multiple indicators can effectively prevent biased judgments originating from reliance on R^2^ alone and reduce the risk of model misjudgment caused by overfitting.

## 3. Results and Discussion

### 3.1. Hyperparameter Tuning Results

To obtain favorable prediction accuracy and generalization performance, this study performs hyperparameter tuning for four machine learning models, namely XGBoost, SVR, GB, and RF, targeting the OMC and MDD, two key soil compaction indicators. The WHO is adopted to search within reasonable parameter ranges, and the optimal hyperparameter combination is determined based on 10-fold cross-validation on the training set.

For tree-based ensemble models, including XGBoost, GB, and RF, this study mainly tunes the number of base estimators, maximum tree depth, learning rate, sampling ratio, and regularization coefficients. These parameters are adjusted to balance the model’s fitting capability and the risk of overfitting. For the SVR model, the kernel function, penalty coefficient C, epsilon-insensitive loss, and kernel coefficient are optimized to improve the nonlinear fitting performance on the soil dataset.

The optimal hyperparameters of each model for the MDD and OMC are summarized in Table 3. Obvious differences can be observed between the parameter configurations of the two prediction tasks. For the XGBoost model, the learning rate for predicting OMC is 0.0798, slightly higher than 0.0746 for MDD, and the number of base estimators varies slightly. The GB model uses a higher learning rate for OMC prediction. When predicting MDD, the RF model uses fewer decision trees with lower maximum depths. The penalty coefficient C and epsilon insensitive loss of the SVR model differ significantly between the two tasks, indicating distinct nonlinear characteristics of the two compaction indicators. A fixed random seed (random_state = 42) is set for all models to ensure the repeatability of experimental results.

### 3.2. Model Performance of MDD Prediction

**Figure 5:**
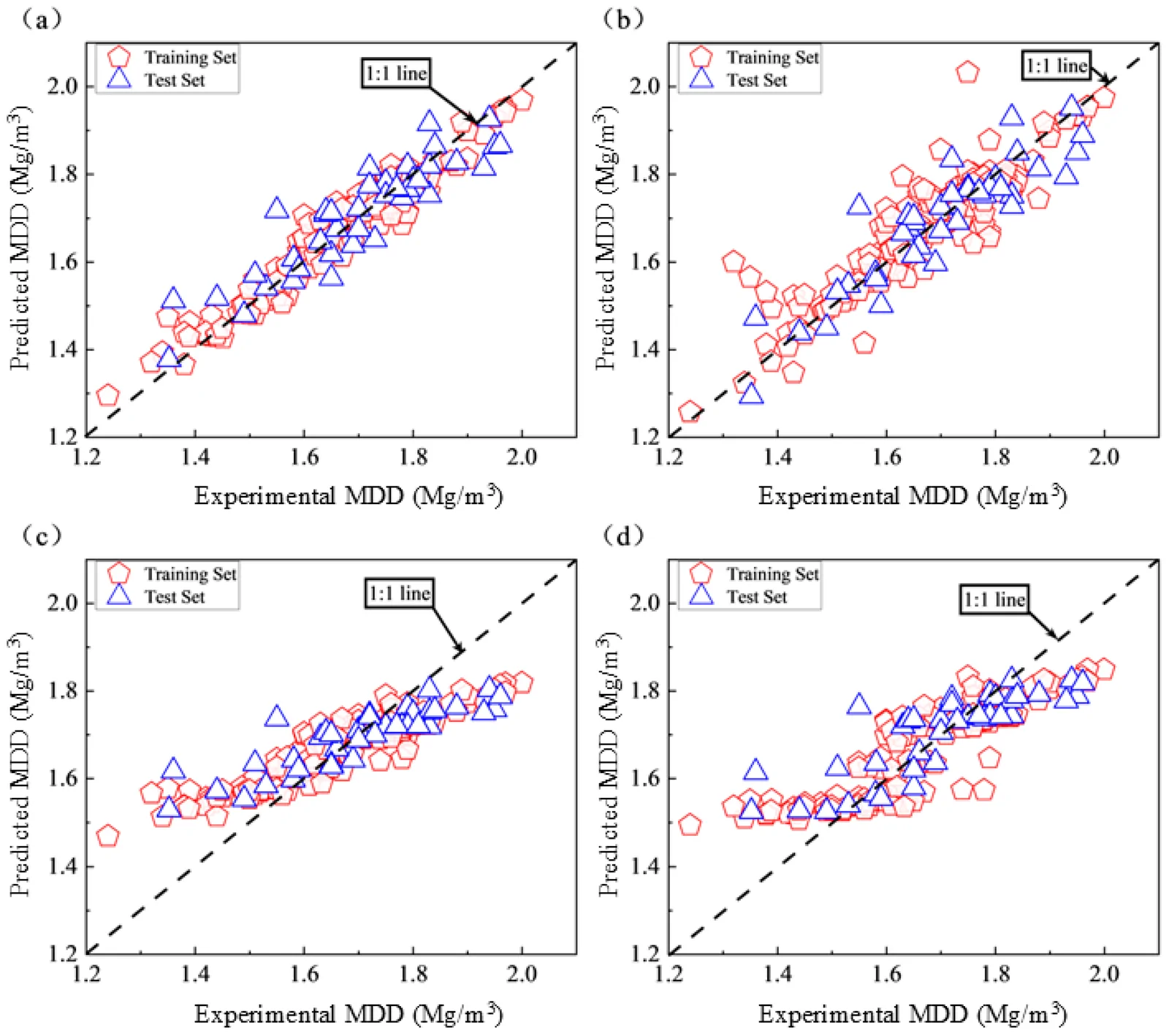
Prediction scatter results of MDD: (**a**) XGBoost; (**b**) SVR; (**c**) GB; and (**d**) RF.

**Figure 6:**
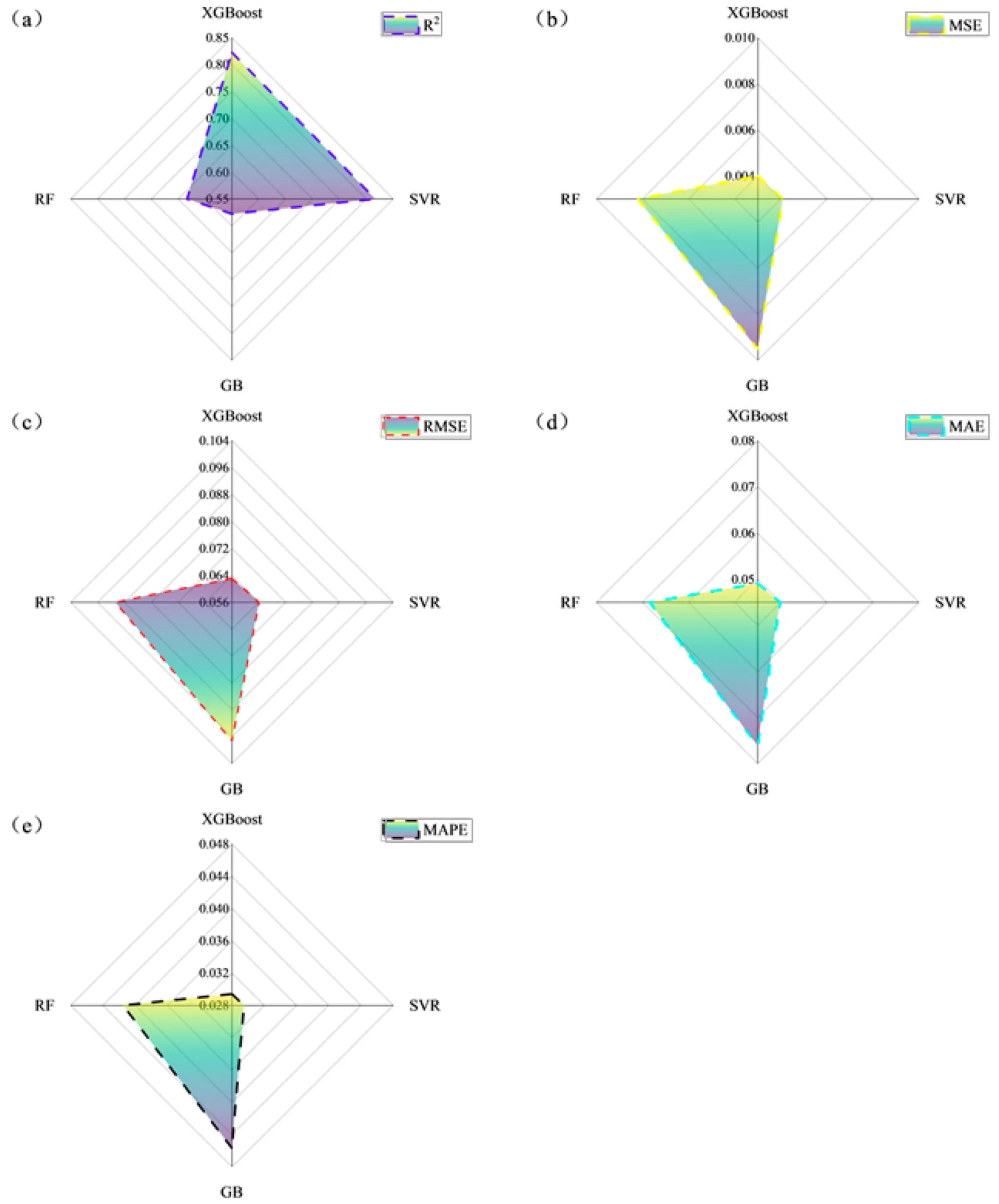
Comparison of prediction performance in test set for MDD with different models: (**a**) R^2^; (**b**) MSE; (**c**) RMSE; (**d**) MAE; and (**e**) MAPE.

This study utilizes a geotechnical dataset with limited samples, where four algorithms, including XGBoost, SVR, GB, and RF, are selected as candidate models for MDD prediction. Figure 5 presents scatter plots of experimental versus predicted MDD values, with the dashed 1:1 diagonal as the ideal prediction reference, while Figure 6 displays radar charts to quantitatively compare five evaluation metrics in test set, R^2^, MSE, RMSE, MAE, and MAPE, for the four models.

Optimized by the Wild Horse Optimizer (WHO), the XGBoost model shown in Figure 5a achieves the best overall predictive performance. Training and test data points are tightly distributed along the 1:1 baseline, without obvious systematic overestimation or underestimation across the full MDD range, and it produces the minimum vertical deviation among all models. This fitting feature is consistent with the quantitative results in Figure 6. WHO-XGBoost occupies the largest R^2^ area and attains the lowest MSE, RMSE, MAE and MAPE values.

The SVR model shown in Figure 5b exhibits acceptable fitting capacity but generates prominent deviations at both low and high MDD intervals, resulting in poorer metric performance than WHO-XGBoost, as reflected in Figure 6. In contrast, GB shown in Figure 5c and RF shown in Figure 5d show severe dispersion of sample points. Numerous specimens, especially those with high MDD, deviate remarkably from the ideal 1:1 line. Correspondingly, Figure 6 shows their low R^2^ and relatively high error metrics, which reveal weak generalization capacity on unseen test samples. The detailed evaluation results of the models are listed in Table 4. Overall, the comparative results demonstrate that WHO-tuned XGBoost outperforms SVR, GB and RF, and it serves as the optimal model for MDD prediction of fine-grained soil.

### 3.3. Model Performance of OMC Prediction

**Figure 7:**
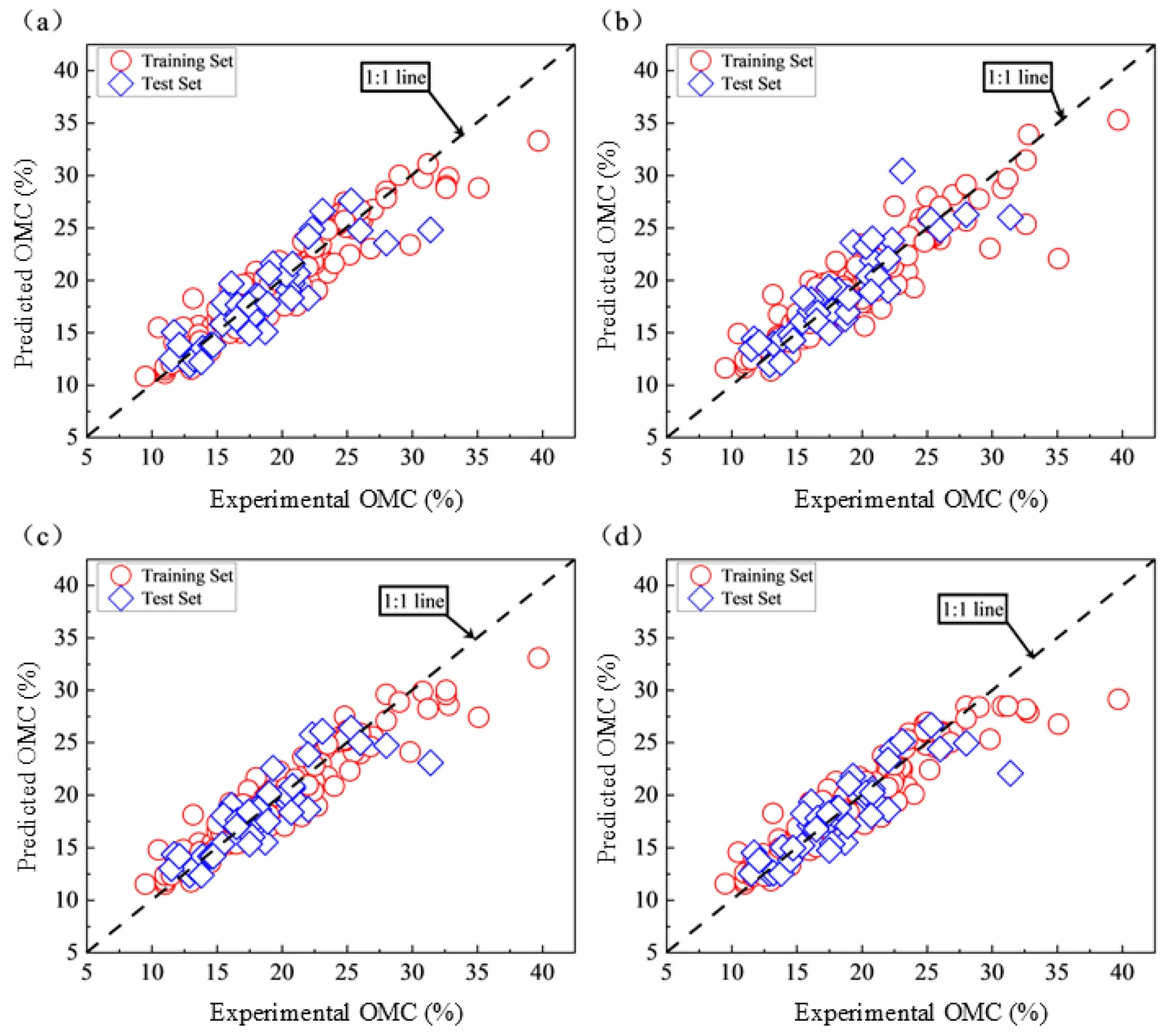
Prediction scatter results of OMC: (**a**) XGBoost; (**b**) SVR; (**c**) GB; and (**d**) RF.

**Figure 8:**
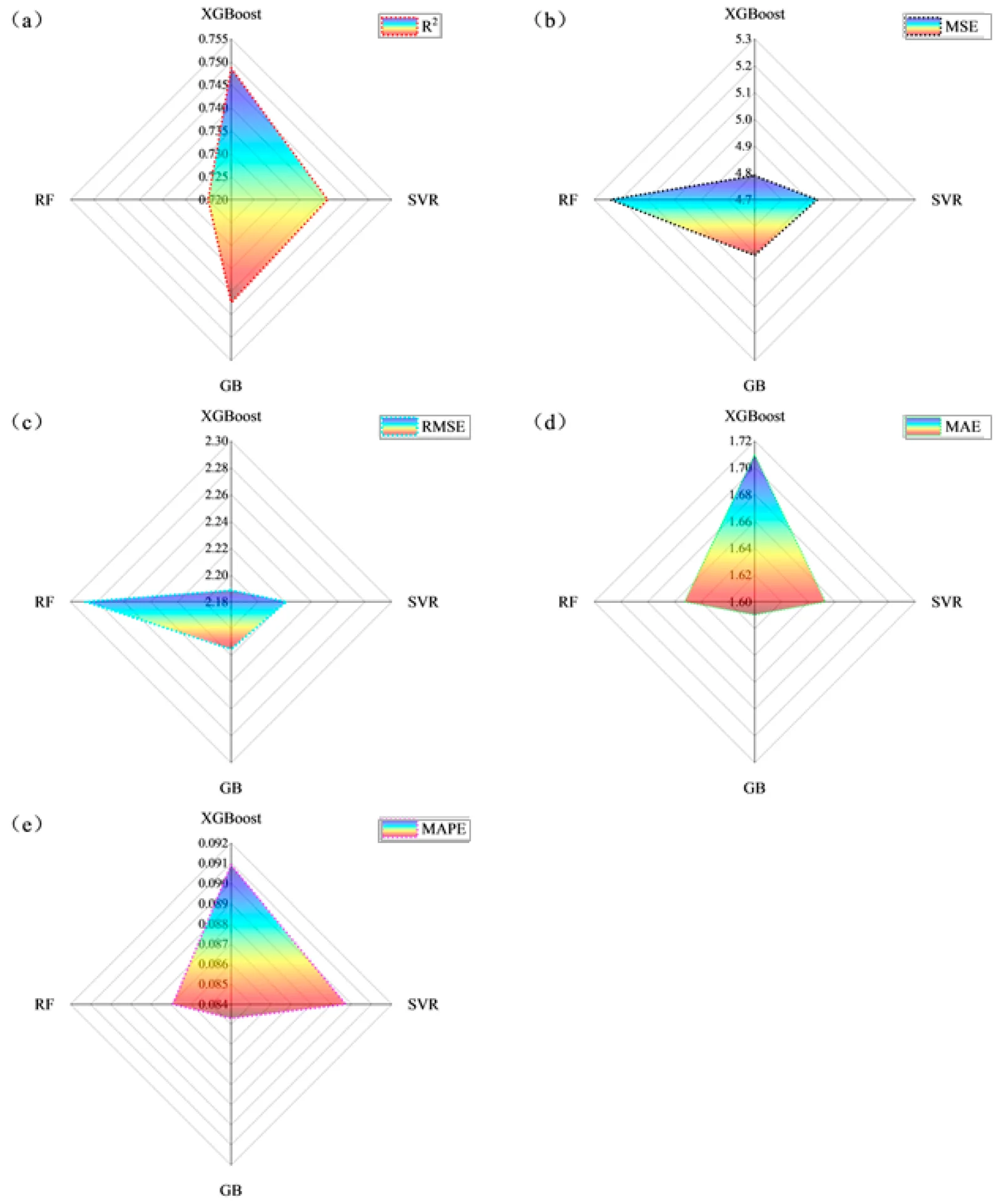
Comparison of prediction performance in test set for OMC with different models: (**a**) R^2^; (**b**) MSE; (**c**) RMSE; (**d**) MAE; and (**e**) MAPE.

Additional model training was carried out to predict the OMC. Figure 7 displays scatter plots of experimental versus predicted OMC with the dashed 1:1 diagonal as the ideal prediction baseline, while Figure 8 provides radar charts comparing R^2^, MSE, RMSE, MAE, and MAPE in test set of the four WHO-tuned models. Compared with the prediction performance of maximum dry density MDD, all four algorithms deliver lower overall accuracy for OMC. This is because the optimum moisture content is more sensitive to the coupled effects of soil plasticity indicators and compaction energy, leading to greater data fluctuations.

Among the candidate algorithms, XGBoost retains the strongest generalization capacity. As shown in Figure 7a, the training and test data points are evenly distributed along the 1:1 reference line; only sparse outliers appear at high OMC, without systematic prediction bias. GB ranks second in comprehensive performance, with stable fitting for moderately deviated samples. SVR achieves a comparable MAPE, yet its R^2^ is slightly lower than GB, and severe point dispersion occurs at both the low and high ends of OMC in Figure 7b. In contrast, RF shows the weakest generalization performance; with large samples, it deviates far from the ideal baseline in Figure 7d, with obvious prediction bias for high OMC soil specimens. The quantitative metric differences are further illustrated in Figure 8, where XGBoost occupies the largest R^2^ region and the smallest error zones, GB achieves competitive R^2^ and the minimum MAE, and RF features the smallest R^2^ area and the maximum error values. The specific prediction performance of each model for OMC is presented in Table 5. Overall, the performance gaps among the four models for OMC prediction are far narrower than those for MDD. Even so, WHO-tuned XGBoost still outperforms SVR, GB and RF in comprehensive prediction stability, further proving its superiority for predicting compaction parameters of fine-grained soil under small-sample conditions.

### 3.4. Sensitivity Analysis

The sensitivity coefficient R_sen_ derived from Equation (9) is used to systematically investigate the sensitivity of soil compaction parameters to various soil physical and chemical properties.

(9) $$R_{\mathrm{sen}}=\frac{\sum_{i=1}^{N}\left(m_{i}t_{i}\right)}{\sqrt{\sum_{i=1}^{N}m_{i}^{2}\sum_{i=1}^{N}t_{i}^{2}}}$$

where m_i_ is the input variable for which the sensitivity analysis is being calculated; t_i_ is the predicted output variable using the proposed prediction model; and N is the number of soil compaction experimental data.

The sensitivity index, influence ranking, and positive or negative correlation between each input feature and MDD and OMC are summarized in Table 6. As shown in Table 6, the sensitivity of soil properties to the two compaction parameters differs considerably.

For MDD, PL has a sensitivity index of −0.7426, indicating a strong negative correlation. Sand content ranks second with a positive sensitivity index of 0.3618. Clay content and LL both exert negative effects, while compaction energy E imposes the weakest positive influence on MDD. For OMC, PL has a high positive sensitivity index of 0.7282. Sand content serves as the secondary negative factor, followed by positively correlated clay content and LL. E exhibits the slightest negative impact on OMC.

### 3.5. SHAP Analysis

**Figure 9:**
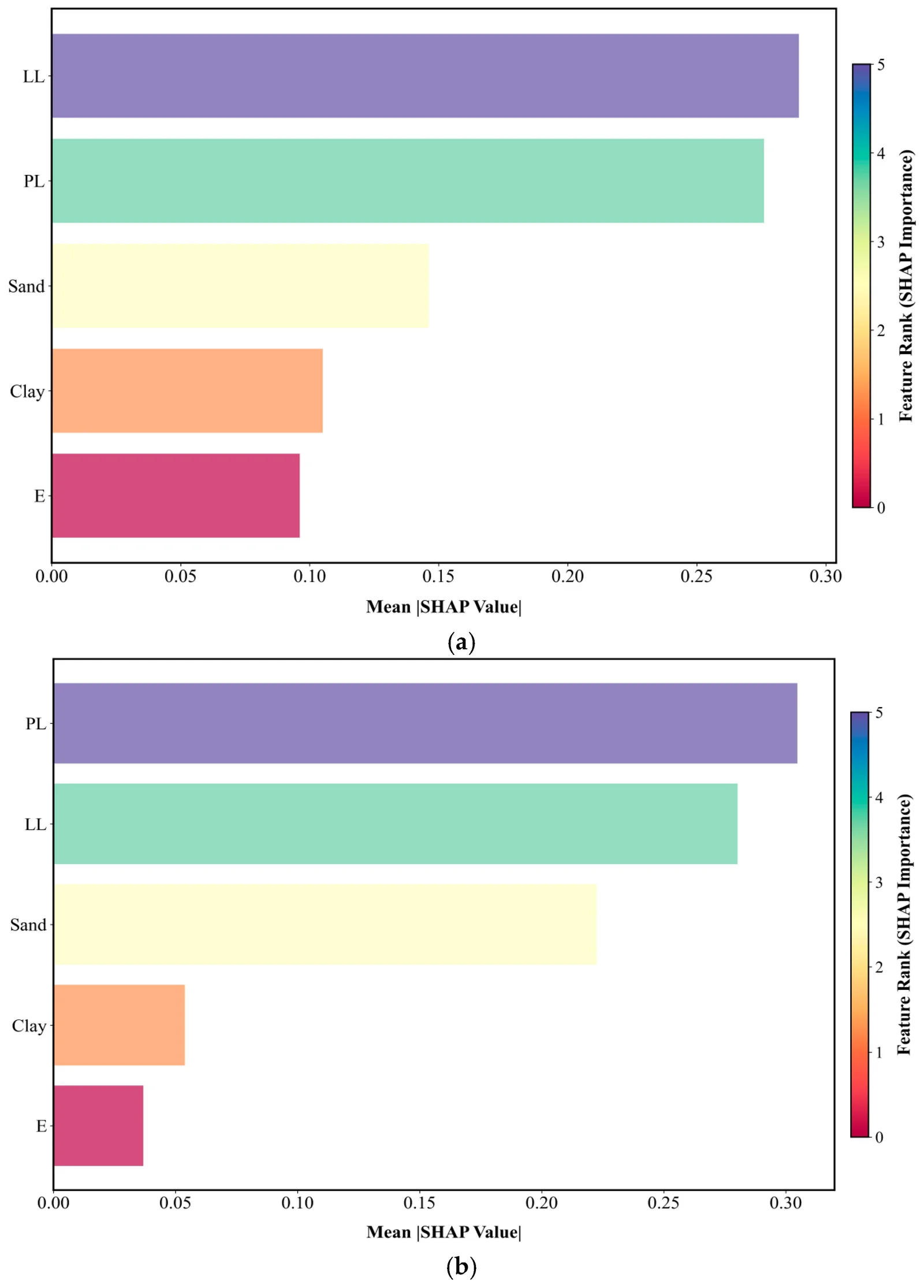
SHAP feature importance of: (**a**) MDD; (**b**) OMC.

To further reveal the influence mechanism of soil properties on soil compaction parameters, the SHAP interpretability analysis was performed on the optimal XGBoost model to explore the model’s internal prediction behavior, and the intrinsic correlation between soil indicators and compaction parameters was interpreted from a machine learning perspective. Figure 9 shows the feature importance ranking for predicting two compaction parameters. In terms of global feature importance, plastic indicators (PL and LL) dominate the prediction of both maximum dry density MDD and OMC, followed by sand and clay fractions, while E exerts the weakest overall effect.

**Figure 10:**
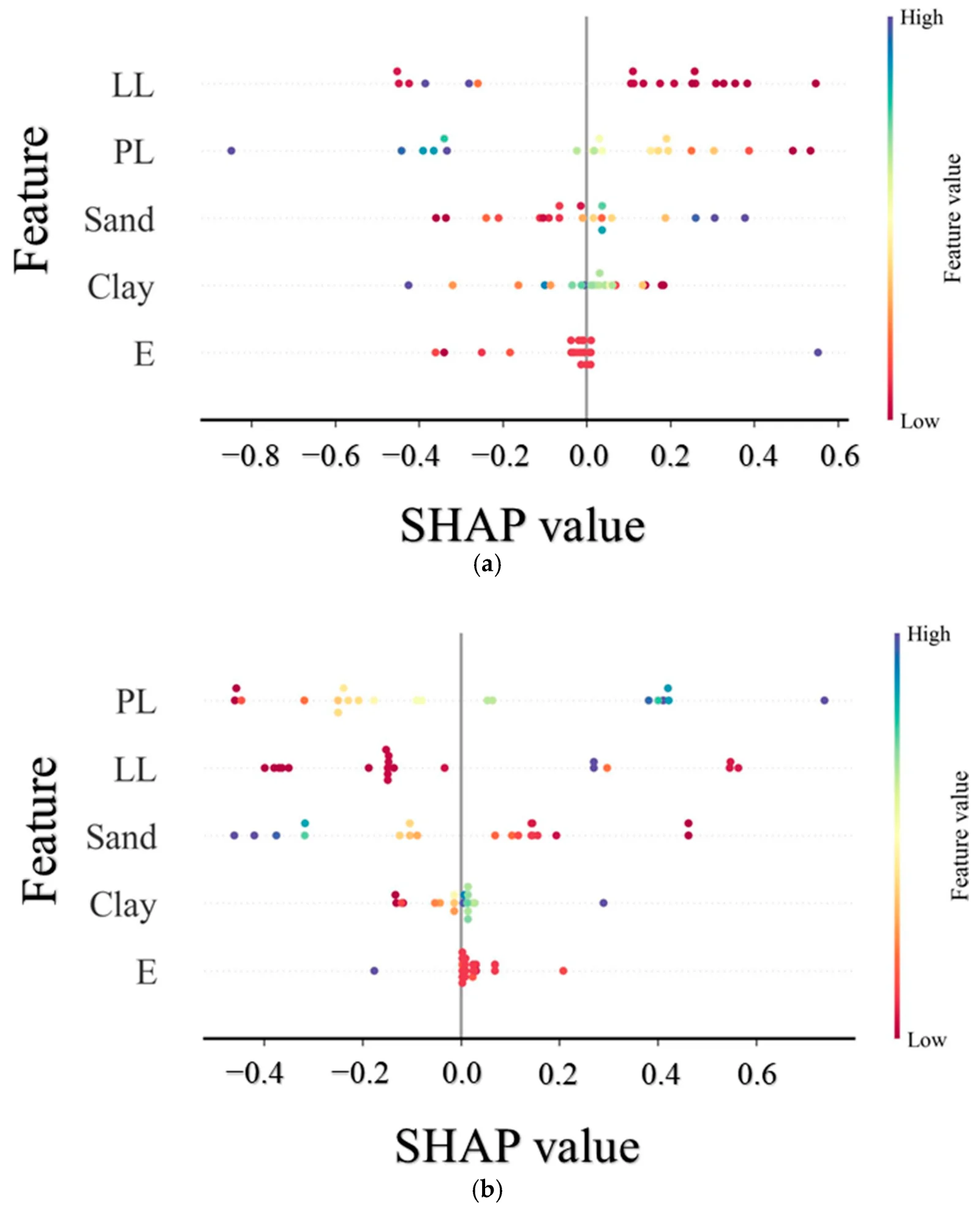
SHAP summary plot of: (**a**) MDD; (**b**) OMC.

Figure 10 shows the SHAP summary plots for predicting MDD and OMC, respectively. The vertical axis denotes input features, and the horizontal axis represents SHAP values. A positive SHAP value indicates that increasing the feature value increases the predicted compaction parameter, while a negative value indicates that increasing the feature value decreases the predicted compaction parameter. The color gradient from red to blue corresponds to low to high feature values. As shown in Figure 10a, for MDD, the SHAP value of LL changes from negative to positive with increasing LL value, meaning high LL contributes to larger MDD. For PL, low feature values correspond to positive SHAP values, while high-value samples yield negative SHAP contributions. This observation is consistent with the inhibitory effect of PL on MDD reported in the sensitivity analysis in Table 6. Sand content shows an obvious nonlinear inflection point as follows: a low sand fraction improves MDD, whereas a high sand fraction reduces it. Clay content consistently exerts a negative influence on MDD, whereas E only produces a minor disturbance. From Figure 10b, PL exhibits a strong positive correlation with OMC. Higher PL leads to a larger OMC. Low LL restrains OMC, while high LL slightly increases it. Sand content positively drives OMC, and clay content first slightly increases and then decreases OMC. E has a weak negative impact on OMC. Comparison of the two plots reveals distinct nonlinear response characteristics of plastic indicators to the two compaction targets, demonstrating differentiated effects of soil plasticity on compaction performance. The overall influence of particle fractions and compaction energy is relatively weak, consistent with the feature importance ranking derived from mean absolute SHAP values. In general, SHAP analysis effectively quantifies the complex, nonlinear coupling between soil physical properties and compaction performance, providing deeper insight into the soil compaction mechanism than linear sensitivity analysis.

### 3.6. Two-Way PDP Analysis

**Figure 11:**
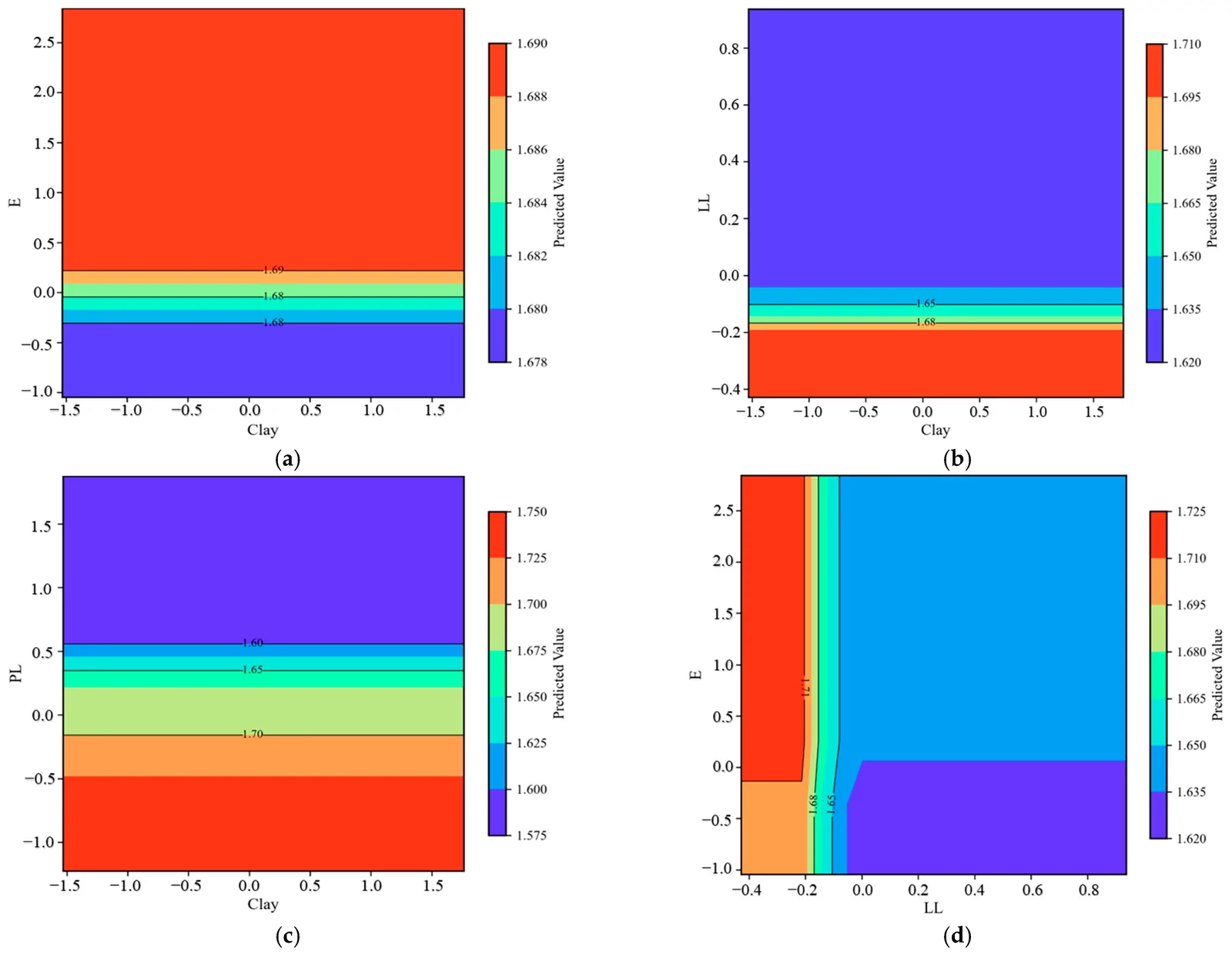

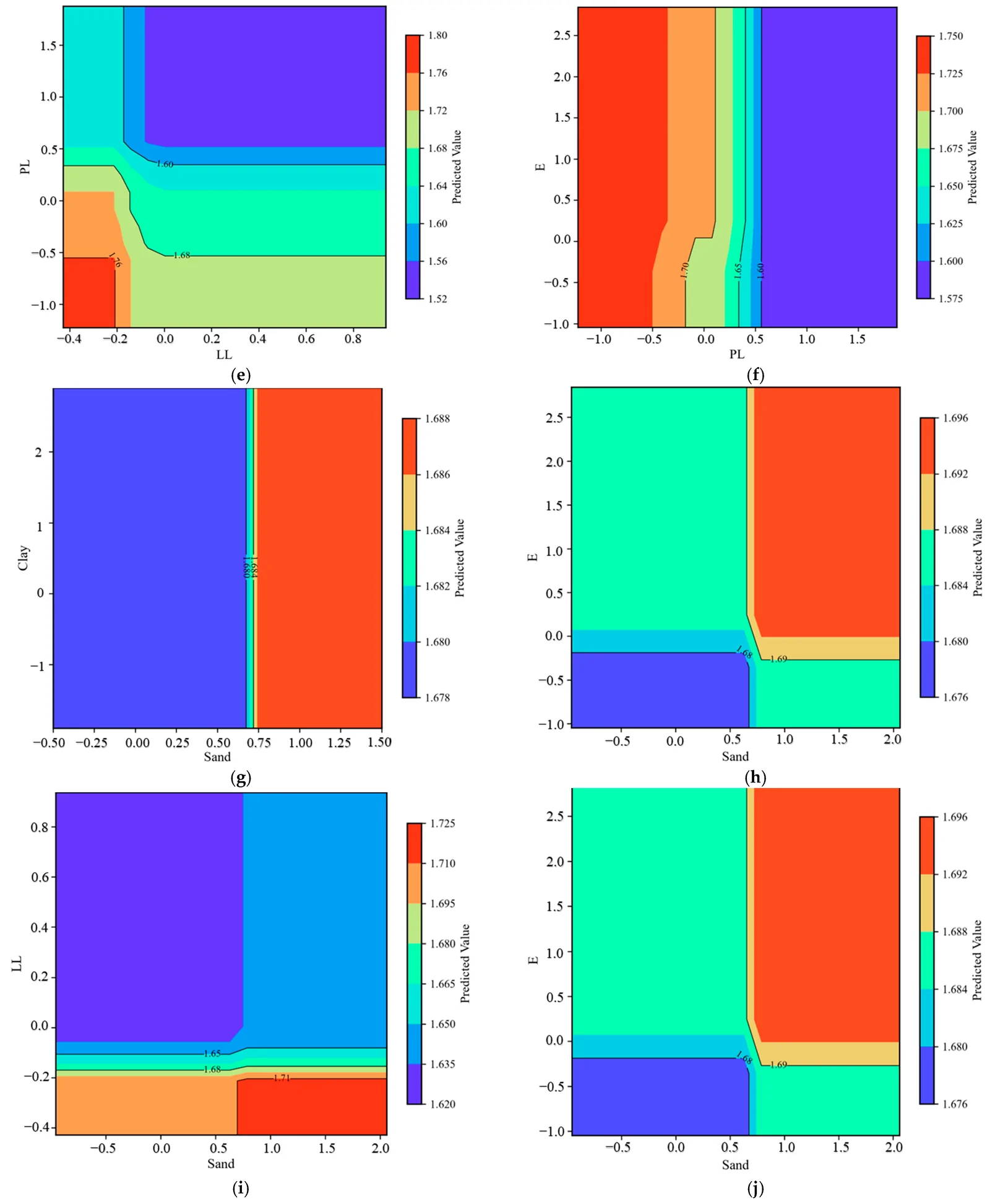
Two-way PDP results for MDD: (**a**) clay and E; (**b**) clay and LL; (**c**) clay and PL; (**d**) LL and E; (**e**) LL and PL; (**f**) PL and E; (**g**) sand and clay; (**h**) sand and E; (**i**) sand and LL; and (**j**) sand and PL.

This study further uses the two-way partial dependence plot (PDP) to visualize the nonlinear coupling effects of two input features on compaction indicators, thereby compensating for the limitation that univariate sensitivity analysis and SHAP values can only reflect independent marginal effects. Figure 11 presents the PDPs of MDD, which visually characterize the nonlinear relationship of MDD under pairwise coupling effects of sand content, clay content, LL, PL and E. The fluctuation amplitude of each surface clearly distinguishes the interaction intensity of feature pairs as follows: surfaces involving PL exhibit the most prominent undulations, followed by LL-related interactions. In contrast, the interaction between the sand and clay fractions is negligible, and all 2D surfaces containing E are generally smooth, with only slight fluctuations, indicating that E shows no remarkable synergistic or antagonistic interactions with basic soil physical indicators. For the surface of LL and PL, the increase in LL continuously reduces MDD under low PL conditions, whereas a slight rise in LL contributes to higher compactness when PL remains at a high level. The global maximum MDD occurs with a combination of high PL and moderate LL, while the minimum dry density occurs with low PL and high LL. A strong negative synergistic effect is observed between clay content and PL; the inhibitory effect of rising PL on MDD becomes more pronounced with increasing clay fraction. Soils with high clay and high PL retain abundant pore water that cannot be fully drained during compaction, leading to severely weakened interparticle interlocking and compaction degree. The interaction between sand and PL follows a piecewise nonlinear pattern. Moderate growth in PL slightly improves compaction performance at low sand content, but MDD declines rapidly as PL increases once sand content exceeds a critical threshold. By comparison, the surfaces of clay and, LL, sand and LL, and sand and clay are monotonous and flat, without inflection points, indicating that particle fractions linearly adjust the MDD without complex nonlinear behavior. Moreover, all E-involved interaction surfaces show negligible variations. Within the compaction energy range of 155–2700 kJ/m^3^ adopted in this study, merely increasing compaction energy can only marginally raise MDD and cannot fundamentally break the compaction ceiling determined by inherent soil plasticity and particle composition, which further verifies that the intrinsic physical properties of soil fill serve as the fundamental factors controlling ultimate compaction performance.

**Figure 12:**
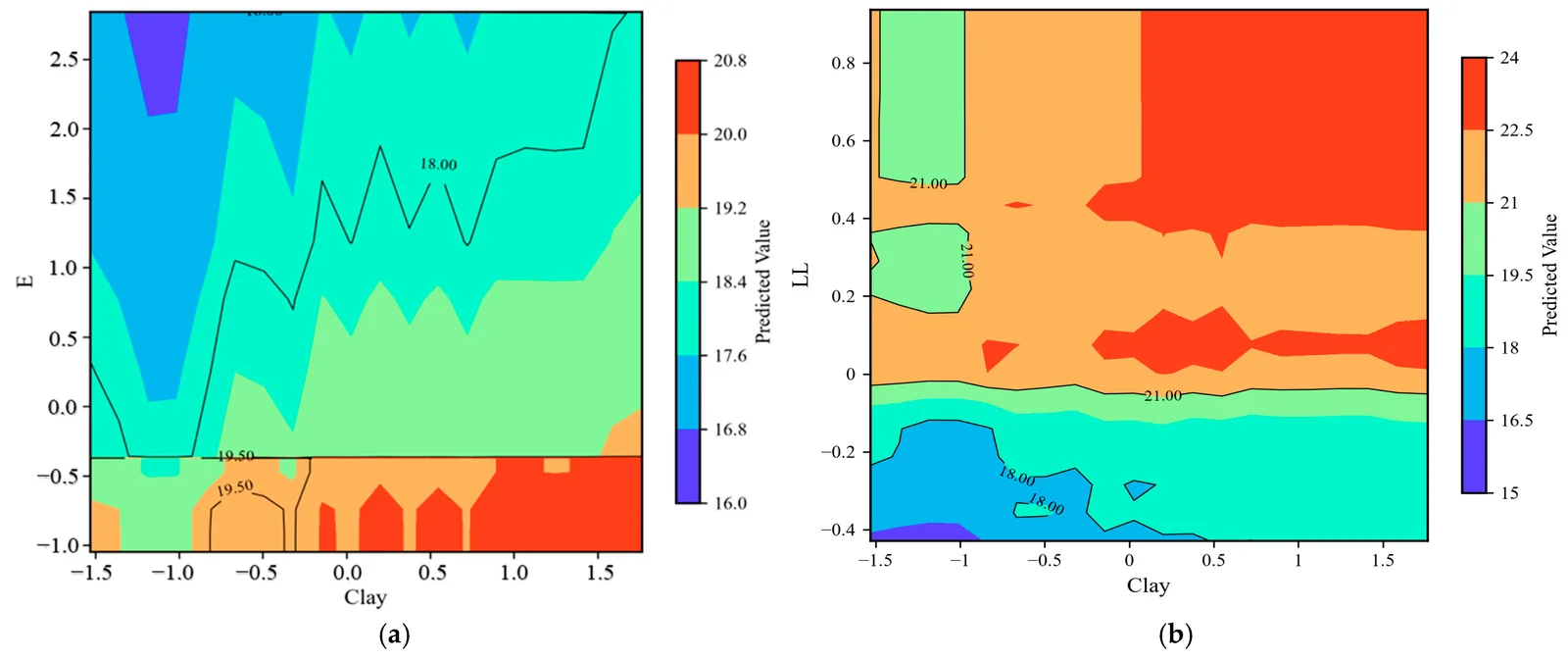

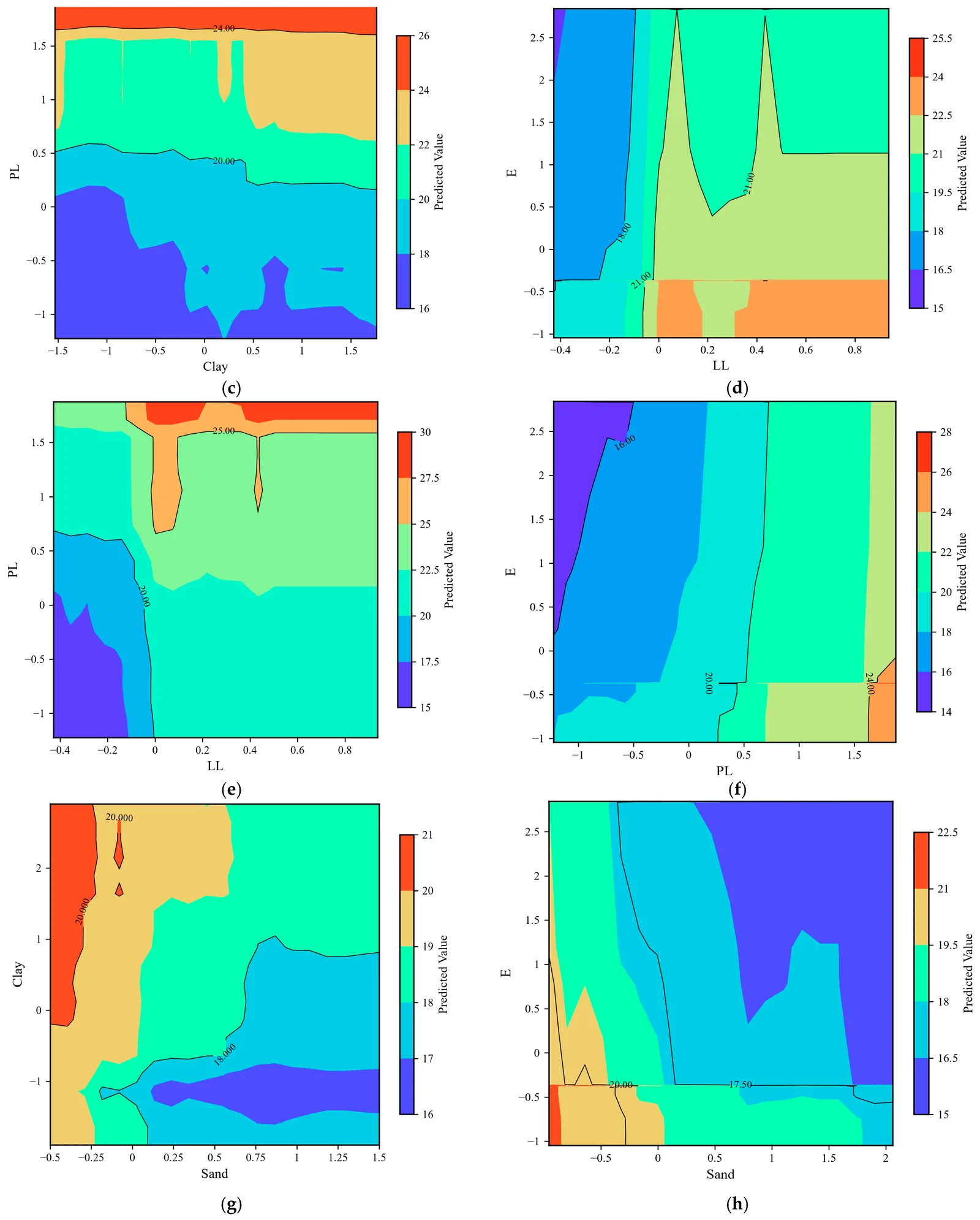

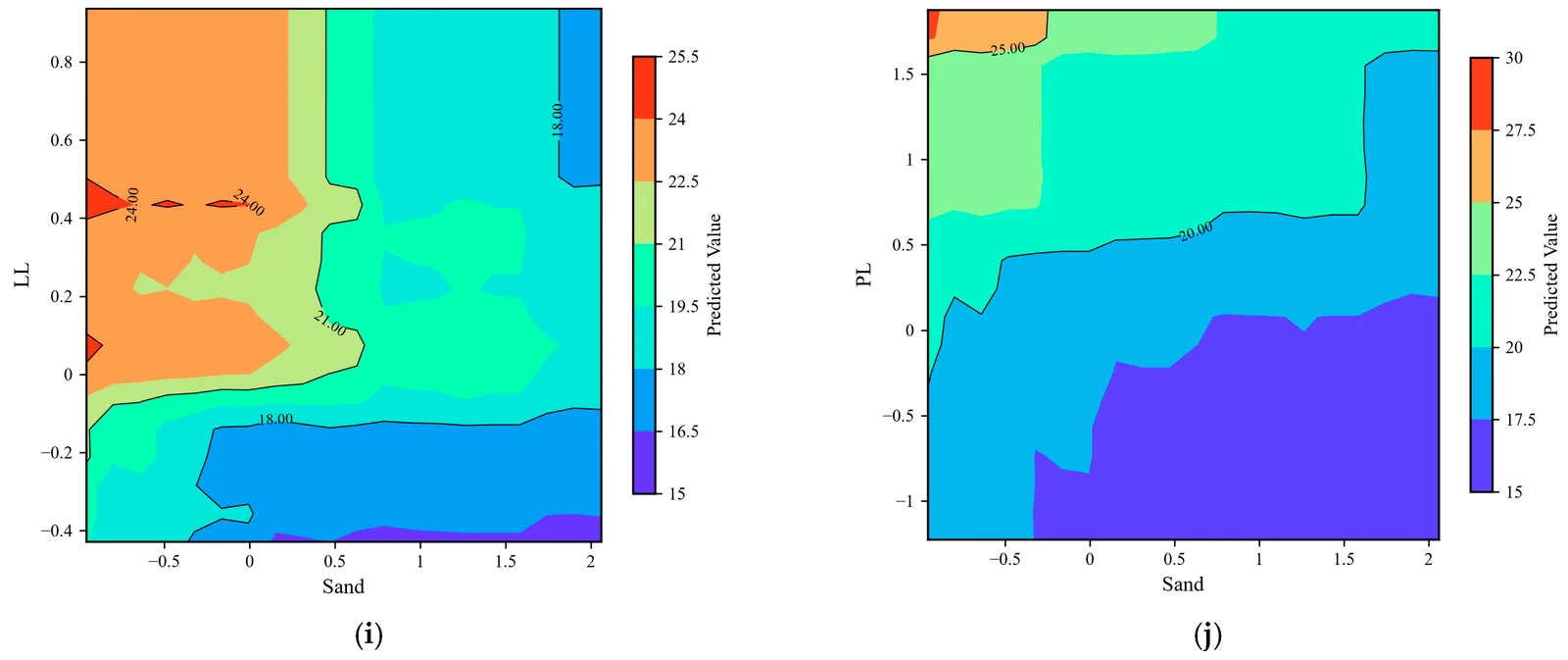
Two-way PDP results for OMC: (**a**) clay and E; (**b**) clay and LL; (**c**) clay and PL; (**d**) LL and E; (**e**) LL and PL; (**f**) PL and E; (**g**) sand and clay; (**h**) sand and E; (**i**) sand and LL; and (**j**) sand and PL.

Figure 12 displays PDPs of OMC, which intuitively reveal the nonlinear variation in OMC under pairwise interactions among sand content, clay content, LL, PL and E. The fluctuation amplitude of each surface clarifies the hierarchy of feature interaction intensity. Surfaces involving plastic limit PL exhibit the most drastic undulations, followed by interactions related to liquid limit LL. The interaction between sand and clay fractions is trivial, and all surfaces incorporating E are generally smooth with minimal fluctuations, indicating that E has no remarkable synergistic or antagonistic interaction with basic soil physical properties. The surface of LL and PL presents prominent positive synergistic characteristics. The growth of PL continuously increases OMC, and the increment of OMC induced by rising LL becomes more significant at higher PL values. The global maximum OMC occurs under the combination of high PL and high LL. Fundamentally, PL determines the soil’s capacity to adsorb stable bound water, and soils with high plasticity contain more active clay minerals, where an elevated liquid limit further increases the water retention demand. A strong positive interaction effect exists between clay content and PL. The promoting effect of increased PL on OMC becomes more prominent with rising clay fraction. Soils with high clay and high PL possess a much larger specific surface area, thus requiring a substantially higher content of pore water for interparticle lubrication. By contrast, the interaction between sand and PL follows a distinct negative pattern. The promotional effect of PL on OMC gradually weakens as the sand proportion increases. Sand-rich soils are dominated by coarse skeleton particles with negligible water adsorption capacity, which counteracts the water retention effect of fine fractions. The surfaces of clay and LL, sand and LL, and sand and clay are monotonous and flat without inflection points, demonstrating that particle fractions only slightly adjust OMC linearly without complex nonlinear coupling behaviors. All E-related interaction surfaces show subtle variations. E exerts a weak negative regulatory effect on OMC overall. Higher E can overcome interparticle frictional resistance by external force and achieve particle interlocking at a lower water content. Nevertheless, the magnitude of OMC reduction induced by elevated E remains nearly constant across the range of particle fractions or plasticity indices, without obvious amplified interaction effects. A comprehensive comparison between Figure 11 and Figure 12 reveals that PL exerts opposite influences on OMC and MDD, visually illustrating the classic geotechnical compaction law that high-plasticity clay soils exhibit high OMC and low MDD.

### 3.7. Model Effectiveness Analysis

As summarized in Table 7, the WHO-XGBoost model proposed in this study exhibits reliable predictive performance for both MDD and OMC compared with three representative hybrid intelligent models from the literature, even when trained on a far smaller dataset (199 samples). The test set R^2^ values of MDD = 0.8226 and OMC = 0.7486 outperform the PSO-XGB, GWO-ANN, and IGWO-ANFIS benchmarks by noticeable margins. These findings demonstrate that the WHO-XGBoost bears research value and practical applicability for geotechnical prediction under small-sample conditions.

### 3.8. Comparison of Hyperparameter Tuning Algorithms

To further explore the influence of hyperparameter optimization strategies on model performance, six hyperparameter tuning algorithms, namely Wild Horse Optimization (WHO), Particle Swarm Optimization (PSO), Gray Wolf Optimizer (GWO), Random Search, Bayesian Optimization and Grid Search, are comprehensively compared for the prediction of MDD and OMC. The comparison metrics cover prediction accuracy, computational overhead, convergence efficiency and the dispersion of optimization results, as summarized in Table 8 and Table 9.

In terms of predictive performance, WHO achieves competitive cross-validation R^2^ and test-set R^2^ for both MDD and OMC tasks. For MDD prediction, Random Search yields the highest mean CV R^2^, closely followed by WHO and GWO. For OMC prediction, WHO obtains the maximum mean CV R^2^. Bayesian Optimization requires the least number of function evaluations but suffers from obvious degradation of generalization performance on the test set, indicating that limited search iterations cannot guarantee reliable optimal hyperparameters for the studied dataset. Heuristic optimization algorithms (WHO, GWO, and PSO) generally deliver better prediction accuracy than traditional Grid Search and Random Search.

From the perspective of computational cost and convergence, PSO and GWO exhibit relatively low time consumption. WHO requires more function evaluations and longer runtime than PSO and GWO but achieves stable and satisfactory predictive accuracy for both targets. In regard to result stability reflected by CV R^2^ IQR and range, Grid Search presents the smallest dispersion for MDD prediction; nevertheless, its overall prediction precision is inferior to heuristic algorithms. WHO maintains moderate result fluctuation and outstanding comprehensive performance for the dual prediction targets of MDD and OMC. Considering prediction accuracy, result stability and practical applicability, the selection of the WHO algorithm for hyperparameter tuning in this work is verified to be reasonable and meaningful. Notably, despite identical preprocessing workflows for the WHO-XGBoost model in both the hyperparameter optimization comparison test and main model development, random train-test data partitioning modifies the sample composition and data distribution of training and test subsets, resulting in minor performance-metric discrepancies (test R^2^ = 0.8226 for MDD, test R^2^ = 0.7486 for OMC in model development; test R^2^ = 0.8429 for MDD, test R^2^ = 0.7759 for OMC in hyperparameter optimization comparison test). This phenomenon is normal and acceptable.

## 4. Conclusions

Based on multi-source geotechnical test data of soil, this study establishes a WHO-tuned machine learning framework for predicting MDD and OMC. Combined with sensitivity and SHAP feature analysis, two-way partial dependence plots are adopted to reveal nonlinear coupling between soil physical indices and compaction behavior, overcoming the limitations of conventional linear regression and single-factor analysis. The main conclusions are summarized as follows:

(1) A standardized dataset of 199 groups of mixed compaction data of soil was constructed after data cleaning. Severe multicollinearity between silt–sand and PI-LL was eliminated by removing silt and PI. The dataset covers soils with wide-ranging particle fractions, liquid/plastic limits and compaction energy, ensuring good model universality.
(2) WHO-optimized XGBoost outperforms SVR, GB, and RF in predicting both compaction parameters. Its test set R^2^ reaches 0.8226 for MDD and 0.7486 for OMC. Benefiting from second-order loss and dual regularization, XGBoost effectively captures nonlinear soil relationships while avoiding overfitting on small datasets. SVR has moderate accuracy, while GB and RF exhibit significant underfitting, especially on high-plastic soils. This verifies the applicability of combining the WHO, a novel hyperparameter tuning algorithm, with XGBoost for predicting soil compaction performance.
(3) PL exerts a considerable influence on compaction performance, followed by sand and clay fractions, whereas E exhibits the weakest effect within the investigated dataset. PL is negatively correlated with MDD and positively correlated with OMC, which agrees with classical geotechnical compaction principles. The SHAP and PDPs interpretation results further uncover nonlinear feature-response relationships that cannot be captured by linear correlation analysis alone.
(4) Two-way PDP analysis reveals strong nonlinear synergistic or antagonistic interactions between PL and LL, whereas particle fractions only produce weak linear adjustments. Within 155–2700 kJ/m^3^, increasing compaction energy slightly improves density but cannot exceed the soil’s inherent compaction limit.
(5) Comparative analyses of WHO, PSO, GWO, Random Search, Bayesian Optimization, and Grid Search on the optimal base model confirm the applicability and feasibility of WHO-XGBoost for the prediction of compacted-soil compaction parameters.

The proposed interpretable framework (WHO-XGBoost-SA-SHAP-PDPs) provides practical engineering value for compaction evaluation of fine-grained soil. It acts as a rapid auxiliary prediction tool for preliminary construction assessment, reducing repetitive Proctor tests and improving construction efficiency. Its interpretable outputs also help engineers identify key soil factors and optimize filling parameters. However, this study has the following limitations: the model is validated only on fine-grained soils using retrospective laboratory data, with uncertain generalization for coarse-grained and modified soils and a lack of prospective field verification. Future work will expand multi-region field datasets, cover broader soil types and compaction conditions, implement external cross-validation, and develop field-oriented intelligent modules for on-site compaction quality assessment.

## Figures and Tables

**Table 1:** Descriptive statistics results.

Variable	Mean	Median	Mode	Standard Deviation	Variance	Minimum	Maximum	Number
Sand (%)	25.57	17.00	2.00	24.65	607.82	0.00	89.00	199
Silt (%)	45.93	52.00	58.00	18.95	358.94	3.00	88.50	199
Clay (%)	28.50	28.00	34.00	14.00	195.93	2.00	69.00	199
LL (%)	54.91	40.00	29.70	68.80	4733.10	20.00	510.00	199
PL (%)	22.32	20.00	18.00	8.38	70.30	6.10	50.00	199
PI	32.58	18.00	10.00	66.43	4412.44	3.40	480.00	199
E (kJ/m^3^)	839.65	593.00	593.00	656.00	430,331.95	155.00	2700.00	199
OMC (%)	19.28	18.20	16.00	4.99	24.91	9.50	39.70	199
MDD (Mg/m^3^)	1.68	1.71	1.75	0.14	0.02	1.24	2.00	199

**Table 2:** Evaluation metrics with ideal values.

Metric	Equation	Ideal Value
R^2^	$R^{2}=1-\frac{\sum_{i=1}^{n}(y_{i}-{\hat{y}}_{i}{)}^{2}}{\sum_{i=1}^{n}(y_{i}-\overset{-}{y}{)}^{2}}$ (4)	1
MAE	$MAE=\frac{1}{n}\sum_{i=1}^{n}|y_{i}-{\hat{y}}_{i}|$ (5)	0
MAPE	$MAPE=\frac{1}{n}\sum_{i=1}^{n}\left|\frac{y_{i}-{\hat{y}}_{i}}{y_{i}}\times 100\right|$ (6)	0%
MSE	$MSE=\frac{1}{n}\sum_{i=1}^{n}(y_{i}-{\hat{y}}_{i}{)}^{2}$ (7)	0
RMSE	$RMSE=\sqrt{\frac{1}{n}\sum_{i=1}^{n}(y_{i}-{\hat{y}}_{i}{)}^{2}}$ (8)	0

Where, y_i_ represents the experimental value for the ith sample, ŷ_i_ represents the model prediction for the ith sample, ȳ represents the average of the actual observations of all samples, and n is the total number of samples, with i ranging from 1 to n.

**Table 3:** Summary of hyperparameter tuning results.

Models	Hyperparameters			
	For MDD Prediction	Value	For OMC Prediction	Value
XGBoost	n_estimators	163	n_estimators	117
	max_depth	4	max_depth	2
	learning_rate	0.0746	learning_rate	0.0798
	min_child_weight	1	min_child_weight	4
	subsample	0.5454	subsample	0.5408
	colsample_bytree	0.5000	colsample_bytree	0.5359
	reg_alpha	0.1115	reg_alpha	0.3000
	reg_lambda	0.5436	reg_lambda	3.0000
	gamma	0.0000	gamma	0.5298
	objective	reg:squarederror	objective	reg:squarederror
	random_state	42	random_state	42
	n_jobs	−1	n_jobs	−1
SVR	C	200.0000	C	51.9381
	epsilon	0.0166	epsilon	1.1154
	kernel	rbf	kernel	rbf
	gamma	0.0100	gamma	0.0632
	degree	3	degree	3
GB	n_estimators	100	n_estimators	64
	max_depth	3	max_depth	3
	learning_rate	0.0100	learning_rate	0.0500
	min_samples_split	2	min_samples_split	10
	min_samples_leaf	1	min_samples_leaf	3
	ccp_alpha	0.0001	ccp_alpha	0.0758
	subsample	0.7000	subsample	0.6032
	max_features	0.5000	max_features	0.4798
	random_state	42	random_state	42
	loss	squared_error	loss	squared_error
RF	n_estimators	50	n_estimators	100
	max_depth	5	max_depth	6
	min_samples_split	8	min_samples_split	8
	min_samples_leaf	1	min_samples_leaf	3
	ccp_alpha	0.0010	ccp_alpha	0.0500
	max_features	0.5000	max_features	0.4439
	random_state	42	random_state	42
	n_jobs	−1	n_jobs	−1

**Table 4:** Results of performance indicators for MDD.

Models	Datasets	R^2^	RMSE	MAE	MAPE	MSE
XGBoost	Training	0.9352	0.0363	0.0286	1.73	0.0013
	Test	0.8226	0.0631	0.0491	2.94	0.0040
SVR	Training	0.8166	0.0625	0.0413	2.53	0.0039
	Test	0.8084	0.0641	0.0499	2.95	0.0041
GB	Training	0.6855	0.0800	0.0629	3.86	0.0064
	Test	0.5777	0.0973	0.0761	4.57	0.0095
RF	Training	0.7207	0.0754	0.0581	3.57	0.0057
	Test	0.6335	0.0907	0.0684	4.15	0.0082

**Table 5:** Results of performance indicators for OMC.

Models	Datasets	R^2^	RMSE	MAE	MAPE	MSE
XGBoost	Training set	0.8792	1.7730	1.2716	6.65	3.1434
	Test set	0.7486	2.1885	1.7096	9.09	4.7894
SVR	Training set	0.8334	2.0824	1.4039	7.31	4.3363
	Test set	0.7410	2.2212	1.6527	8.97	4.9338
GB	Training set	0.8786	1.7771	1.2910	6.77	3.1581
	Test set	0.7424	2.2153	1.6097	8.47	4.9078
RF	Training set	0.8687	1.9176	1.2976	6.66	3.6772
	Test set	0.7250	2.2890	1.6522	8.69	5.2394

**Table 6:** Sensitivity analysis results regarding the effect of the input variables on the output variables.

Compaction Parameter	Feature	Sensitivity Index	Rank	Correlation
MDD	PL (%)	−0.7426	1	Negative
	Sand (%)	0.3618	2	Positive
	Clay (%)	−0.3461	3	Negative
	LL (%)	−0.2755	4	Negative
	E (kJ/m^3^)	0.2003	5	Positive
OMC	PL (%)	0.7282	1	Positive
	Sand (%)	−0.5238	2	Negative
	Clay (%)	0.4769	3	Positive
	LL (%)	0.2197	4	Positive
	E (kJ/m^3^)	−0.167	5	Negative

**Table 7:** Research comparison.

Related Research	Data Size	Test R^2^ of MDD (Best Model)	Test R^2^ of OMC (Best Model)
Verma et al. (2023) [12]	2148	0.674 (PSO-XGB)	0.635 (PSO-XGB)
Bardhan and Asteris (2023) [57]	532	0.71 (GWO-ANN)	0.73 (GWO-ANN)
Bardhan et al. (2023) [10]	251	0.7428 (IGWO-ANFIS)	0.7473 (IGWO-ANFIS)
This study	199	0.8226 (WHO-XGB)	0.7486 (WHO-XGB)

**Table 8:** Comparison results of hyperparameter tuning methods for MDD.

Methods	CV R^2^ Mean	Test R^2^	Time (s)	Num Evals	CV R^2^ Std	CV R^2^ Range	CV R^2^ IQR
WHO	0.7717	0.8429	31.5810	240.000	0.0572	0.1697	0.0540
PSO	0.7594	0.8221	14.6988	168.000	0.0635	0.1715	0.1012
GWO	0.7740	0.8189	14.7687	168.000	0.0629	0.1779	0.0698
Random Search	0.7775	0.8031	19.2488	200.000	0.0559	0.1595	0.0654
Bayesian Optimization	0.7703	0.7905	43.6495	60.000	0.0649	0.1770	0.0949
Grid Search	0.7561	0.7758	21.6222	200.000	0.0279	0.0719	0.0414

**Table 9:** Comparison results of hyperparameter tuning methods for OMC.

Methods	CV R^2^ Mean	Test R^2^	Time (s)	Num Evals	CV R^2^ Std	CV R^2^ Range	CV R^2^ IQR
WHO	0.7766	0.7759	21.1320	240.0000	0.0598	0.1473	0.1141
Random Search	0.7756	0.7672	15.6405	200.0000	0.0569	0.1611	0.0785
Grid Search	0.7714	0.7602	15.6573	200.0000	0.0516	0.1355	0.0789
GWO	0.7737	0.7528	13.7754	168.0000	0.0532	0.1452	0.0846
PSO	0.7658	0.7306	13.3848	168.0000	0.0726	0.2028	0.1061
Bayesian Optimization	0.7646	0.7030	39.8633	60.0000	0.0644	0.1573	0.1280

## Data Availability

The original contributions presented in this study are included in the article. Further inquiries can be directed to the corresponding author.
